# Paternal Poly (ADP-ribose) Metabolism Modulates Retention of Inheritable Sperm Histones and Early Embryonic Gene Expression

**DOI:** 10.1371/journal.pgen.1004317

**Published:** 2014-05-08

**Authors:** Motomasa Ihara, Mirella L. Meyer-Ficca, N. Adrian Leu, Shilpa Rao, Fan Li, Brian D. Gregory, Irina A. Zalenskaya, Richard M. Schultz, Ralph G. Meyer

**Affiliations:** 1 Department of Animal Biology and Center for Animal Transgenesis and Germ Cell Research, School of Veterinary Medicine, University of Pennsylvania, Philadelphia, Pennsylvania, United States of America; 2 Department of Biology, School of Arts and Sciences, University of Pennsylvania, Philadelphia, Pennsylvania, United States of America; 3 School of Medicine, Microarray Core Facility, University of Pennsylvania, Philadelphia, Pennsylvania, United States of America; 4 School of Arts and Sciences, Penn Genome Frontiers Institute, University of Pennsylvania, Philadelphia, Pennsylvania, United States of America; 5 CONRAD, Department of Obstetrics and Gynecology, Eastern Virginia Medical School, Norfolk, Virginia, United States of America; University of Texas at San Antonio, United States of America

## Abstract

To achieve the extreme nuclear condensation necessary for sperm function, most histones are replaced with protamines during spermiogenesis in mammals. Mature sperm retain only a small fraction of nucleosomes, which are, in part, enriched on gene regulatory sequences, and recent findings suggest that these retained histones provide epigenetic information that regulates expression of a subset of genes involved in embryo development after fertilization. We addressed this tantalizing hypothesis by analyzing two mouse models exhibiting abnormal histone positioning in mature sperm due to impaired poly(ADP-ribose) (PAR) metabolism during spermiogenesis and identified altered sperm histone retention in specific gene loci genome-wide using MNase digestion-based enrichment of mononucleosomal DNA. We then set out to determine the extent to which expression of these genes was altered in embryos generated with these sperm. For control sperm, most genes showed some degree of histone association, unexpectedly suggesting that histone retention in sperm genes is not an all-or-none phenomenon and that a small number of histones may remain associated with genes throughout the genome. The amount of retained histones, however, was altered in many loci when PAR metabolism was impaired. To ascertain whether sperm histone association and embryonic gene expression are linked, the transcriptome of individual 2-cell embryos derived from such sperm was determined using microarrays and RNA sequencing. Strikingly, a moderate but statistically significant portion of the genes that were differentially expressed in these embryos also showed different histone retention in the corresponding gene loci in sperm of their fathers. These findings provide new evidence for the existence of a linkage between sperm histone retention and gene expression in the embryo.

## Author Summary

That not all histones are replaced by protamines in the sperm nucleus during spermiogenesis has been known for almost three decades, along with the notion that protamines do not bear any specific epigenetic information whereas histones typically carry posttranslational modifications with epigenetic regulatory functions. The enrichment of histones with distinct epigenetic modifications around transcriptional start sites, as well as unmethylated GC-rich promoter regions and exons in murine and human sperm, has recently been demonstrated by others at high resolution. The evolutionary conservation of the common principles underlying sperm histone retention provides a plausible rationale for epigenetic inheritance by nucleosomes. The present study takes a different approach towards testing the overarching hypothesis that sperm histones are linked to early embryonic gene expression by analyzing expression of genes in 2-cell embryos originating from sperm in which gene histone association of these genes was experimentally altered. The results are consistent with the aforementioned hypothesis and support the view of sperm histones as potential mediators of epigenetic inheritance through the male germ line, which could also contribute to phenotypic variation in mammals in response to environmental or dietary factors that affect sensitive chromatin-modulating pathways such as PAR metabolism.

## Introduction

During gametogenesis, male and female germ cells are epigenetically reprogrammed as they undergo sex-specific differentiation into functional gametes. After fertilization, chromatin in male and female pronuclei continues to be epigenetically remodeled prior to the first round of DNA replication and first cleavage. These changes include changes in the complement of histones associated with DNA as well as DNA demethylation. The first major wave of genome activation in mice occurs during the two-cell embryo stage and epigenetic reprogramming continues until the blastocyst stage (reviewed e.g. in [1]).

**Figure 1:**
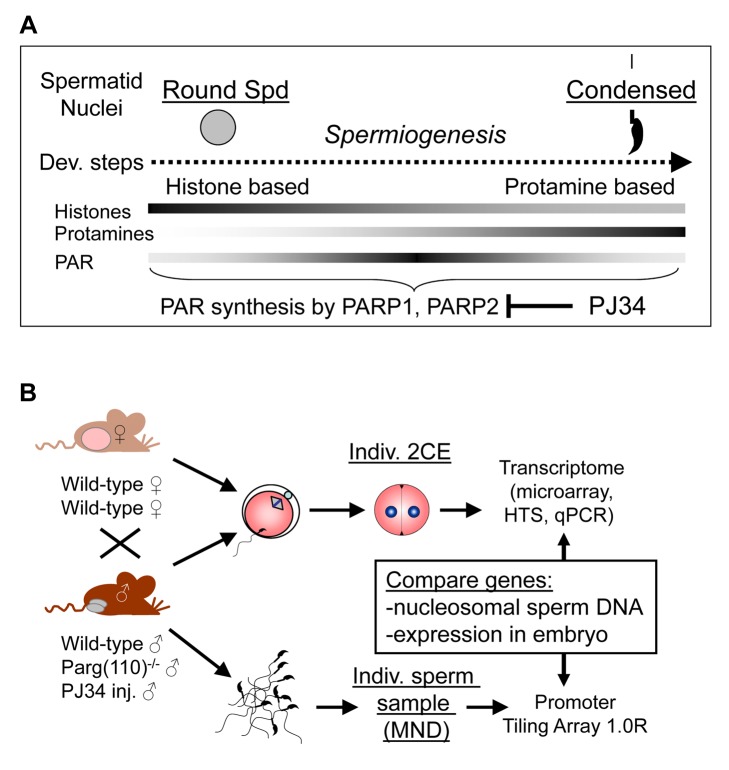
Experimental design to ascertain the impact of sperm chromatin structure on early embryonic gene expression. (A) Efficiency of histone-to-protamine exchange in spermiogenesis depends in part on levels of poly(ADP-ribose) (PAR) formed transiently by the interplay of PAR polymerases (PARP1, PARP2) and PAR glycohydrolase (PARG). Inhibition of PAR synthesis by PJ34 or disruption of normal PARG activity in the *Parg*(110)^−/−^ mouse leads to abnormal chromatin remodeling with retention of histones in sperm [29]. (B) Natural mating of *Parg*(110)^−/−^ males or males treated with PJ34 with wild-type control females was used to obtain 2-cell stage embryos (2CE) for genome-wide transcriptional profiling at the individual embryo level using microarrays and high throughput sequencing (HTS). Cauda epididymal sperm from the fathers were used to identify genes associated with nucleosomes rather than protamines using micrococcal nuclease digestion (MND). Aberrant histone association of gene loci with differential expression of genes in two-cell embryos was assessed and compared to embryo expression data.

Immediately after fertilization, the differential remodeling of maternal and paternal chromatin likely reflects differences in chromatin composition of the gametes that is acquired during gametogenesis. During the post-meiotic steps of spermatogenesis, termed spermiogenesis, the haploid germ cells (spermatids) undergo dramatic chromatin remodeling that entails replacement of most histones with small, highly basic protamines that facilitate extensive condensation of sperm nuclei (Fig. 1A) [2], [3]. In contrast to maternal chromatin, whose DNA remains packaged in nucleosomes, paternal chromatin following fertilization undergoes a rapid decondensation of the compact sperm head and re-establishment of nucleosomal chromatin by replacement of protamines with maternally-derived histones.

Mature, condensed sperm nuclei contain only residual amounts of nucleosomes, approximately 1% in the mouse, that are preferentially located at specific sites within the genome, e.g., genes associated with development and cell signaling [4]–[7], centromeric and telomeric domains [8], retroposon DNA [9], and CCCTC factor (CTCF)-binding sites [4]. The functional consequences of such association of residual histones with specific promoter regions in regulating embryonic gene expression could, in principle, influence gene expression in the embryo by marking these promoters [10]–[12]. Recent studies have used chromatin immunoprecipitation with histone and histone modification- specific antibodies in combination with DNA deep sequencing to map histone association in human and murine sperm at high resolution [5], [7], [13]. In human, retained modified sperm histones, such as histone H3 dimethylated at lysine 4 (H3K4me2), an activating mark, are enriched at specific developmental promoters, and trimethylated H3K4 (H3K4me3) is found at paternally-expressed imprinted loci, microRNA genes, HOX gene clusters and other genes [7]. In contrast, the repressive histone mark H3K27me3 is enriched in promoters of genes that are important for development but not expressed during preimplantation embryo development [7]. In mice, the retained nucleosomes are enriched in GC-rich but unmethylated promoter regions and are largely composed of the histone H3.3 variant, regardless whether they carry H3K4 or H3K9 trimethylation marks [5], [13]. The enrichment of nucleosomes retained at promoters with the histone variant H3.3 correlates with post-meiotic gene activity in round spermatids prior to nuclear condensation and H3K4 methylation marks but not H3K27 methylation [13].

There is evidence that retained sperm histones remain associated with the paternal genome after fertilization [14]. Apart from the sites that had retained histones in sperm, however, the majority of the genome in the decondensing male pronucleus becomes associated with hypomethylated maternal histones during the post-fertilization period of protamine-histone exchange. The female pronucleus, on the other hand, is enriched with methylated H3K4 (H3K4me) [15], as well as di- and trimethylated H3K9 and H3K27 (H3K9me2, H3K9me3, H3K27me3), which are nearly absent from the male pronucleus that contains only monomethylated H3K9 [16]–[18]. The resulting asymmetrical reprogramming of the two parental pronuclei could provide a platform for differential transcription of the paternal and maternal genomes that reside in the same nucleus starting at the 2-cell stage. In summary, modified histones retained in the sperm according to common principles conserved in mouse and human could represent an epigenetic signature that becomes recognized during embryonic development [13], [19], [20].

There are further clues that such an epigenetic signature in sperm could regulate gene expression in the preimplantation embryo. Whereas DNA in the female pronucleus is largely protected from demethylation by maternal factors such as PGC7/STELLA [21], paternal DNA is extensively demethylated except for certain domains such as imprinted genes that somehow escape demethylation [22]. Potentially, maternal, and perhaps paternal, DNA could also be protected from demethylation by repressive histone marks, e.g., H3K9me3 and H3K27me3 [17], [23]. These differences in DNA methylation ultimately are linked to parent-of-origin differences in gene expression in the embryo [24], [25].

The present study used an alternative approach to test the overarching hypothesis that retained sperm histones are informative to early embryo gene expression by tracking the expression of genes in early embryos originating from males in which histone association in the corresponding sperm loci of these genes was experimentally altered. We previously reported that chromatin remodeling, and in particular the exchange of histones for protamines during spermiogenesis, is facilitated by the activity of poly(ADP-ribose) polymerases PARP1 and PARP2 [26], [27] (Fig. 1A). These enzymes produce poly(ADP-ribose) (PAR) in response to DNA strand breaks that naturally occur during spermiogenesis; the newly synthesized PAR is rapidly degraded by poly(ADP-ribose) glycohydrolase (PARG). The pathway affords transient local chromatin decondensation, and interfering with PAR synthesis or degradation during mouse spermiogenesis results in abnormal chromatin structure with reduced density of chromatin packaging and elevated levels of retained histones [28], [29]. We used either genetic disruption of the *Parg* gene or pharmacological inhibitors of PARP enzymes to alter PAR metabolism in males. Of relevance, no residual PARP, PARG or PAR is detectable in mouse sperm, which have completed chromatin remodeling [28].

To assess the effect of histones retained in sperm on gene expression in the early embryo, the locations of abnormally retained histones in sperm from individual mice with perturbed PAR metabolism were mapped, and gene expression in single embryos fathered by these males was analyzed (Fig. 1B). We report that perturbing sperm chromatin composition by altering PAR metabolism in male mice leads to differential gene expression during the maternal-to-embryo transition in individual progeny 2-cell embryos derived from crosses with wild-type females. Strikingly, and unexpectedly, a highly significant correlation is observed between the aberrant retention of histones in sperm promoter regions and differential expression of these same affected genes in 2-cell embryos. The data provide new evidence that sperm histones confer epigenetic information to the zygote that regulates transcription in the 2-cell embryo. The findings also suggest that pharmacological alteration of a paternal metabolic pathway (and therefore environmental perturbations) has the potential to change gene expression in embryos fathered by these males through modulation of sperm chromatin composition.

## Results

### Altering PAR metabolism causes abnormal sperm histone retention

*Parg*(110)^−/−^ mice have nearly normal sperm morphology and motility but significantly reduced litter sizes [28]. Spermatozoa from these mice are characterized by reduced nuclear condensation, as indicated by increased chromomycin A3 (CMA3) staining [29]. CMA3 is a fluorescent dye that intercalates into DNA, but not in protaminated sperm DNA, which is too densely packed. The degree of CMA3 staining of sperm thus provides a measure of relative protamine deficiency in individual nuclei due to increased histone retention [30].

**Figure 2:**
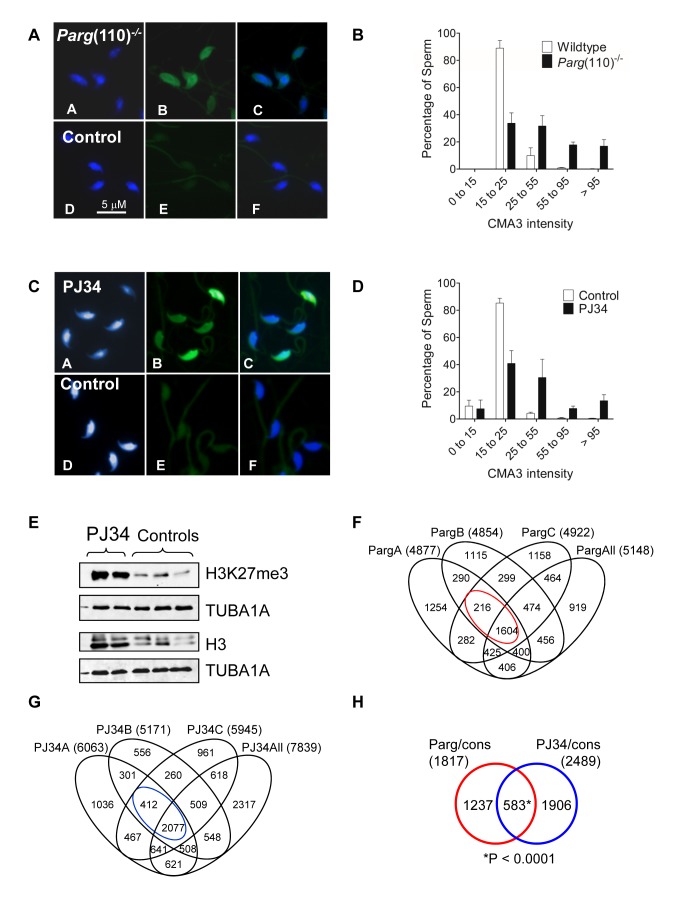
Aberrant chromatin composition in mouse models of altered PAR metabolism. Chromomycin A3 (CMA3) intercalation into the DNA indicates incomplete chromatin condensation in *s*perm from *Parg*(110)^−/−^ (A) and PJ34-treated (C) males with histone retention. (B, D) Histogram of sperm CMA3-staining intensities reflects that severity of CM3A staining varied at the level of individual sperm and individual fathers (n>200 nuclei/sample, 3 males/group). (E) Immunoblot analyses of sperm protein lysates showing increase in histone retention in PJ34 treated males. TUBA1A: alpha tubulin loading control. (F) Overlaps of genes identified as differentially histone associated in sperm from 3 individual *Parg*(110)^−/−^ males (“PargA”, “PargB”, “PargC”, the fathers of the embryos analyzed below) by micrococcal nuclease digests (MND) compared to the wild-type controls. The “PargAll” data set contains all genes commonly identified as differentially MNase-sensitive across 10 *Parg*(110)^−/−^ males compared with 9 wild-type control animals. The red circle indicates common genes that were differentially histone associated in all groups (1604+216 = 1820, red circle) compared with wild-type. (G) PJ34: differentially MNase-sensitive genes in three different males (like in E) and overlap with a surrogate dataset (“PJ34All”) consisting of data from all 4 PJ34-treated males compared with 9 wild-type control males. The overlap of 2,489 genes that were commonly differentially histone associated in sperm samples is indicated (blue circle). (H) Overlap of genes commonly affected by differential histone association between the *Parg*(110)^−/−^ and the PJ34 models compared to wild-type controls (red and blue circles in F and G). A Pearson correlation examining significance of this overlap using a genetic background of 19,472 genes was calculated with a resulting P<0.0001, dismissing the null hypothesis that the observed overlap is coincidental (predicted number). The list and GO-term analysis of the 583 genes is contained in Dataset S4 (MS Excel).

Quantification of CMA3 staining showed that the *Parg*(110)^−/−^ sperm populations from the sires used in this study exhibited a wide range of elevated staining intensities above background (Fig. 2A, B), indicating elevated nucleosome retention in the majority of individual sperm cells. These differences are in accord with the elevated retention of core histones, testis-specific histone variants including TH2B, H1T and HILS1, as well as macroH2A in mature sperm from such animals as detected previously by immunoblot analyses [29]. We reported earlier that histone H3, which is retained at two- to three-fold higher levels in *Parg*(110)*^−/−^* sperm, also bear activating or repressive modifications, e.g., H3K4me3, H3K9me2, H3K9me3, H3K27me3 [29].

In addition to the *Parg*(110)^−/−^ mice, a second, pharmacological animal model of perturbed PAR metabolism was used. In this model we induced excessive histone retention by injecting wild-type males with PJ34, a potent inhibitor of PARP1 and PARP2, for six weeks prior to mating these males to wild-type females of the same inbred strain (129SVE, Taconic). These animals appear completely normal during and after treatment with the exception of the formation of an abnormal sperm chromatin structure that closely resembles the *Parg*(110)^−/−^ phenotype (Fig. 2C, D), an effect we previously described [29]. Of note, in both mouse models not all sperm are equally affected by elevated histone retention and ∼25% (*Parg*(110)^−/−^) to 35% (PJ34-injected) sperm had only weak CMA3 fluorescence similar to most sperm present in wild-type (Fig. 2B, D). In summary, these data confirmed our previous observations that perturbing PAR metabolism results in elevated levels of histone retention and reduced chromatin density. Similar to *Parg*(110)^−/−^ mice, histones retained in sperm from PJ34-injected mice include testis-specific variants, such as TH2B and HILS1 [29], and the retained histones bear epigenetically relevant tail modifications such as H3K27me3 (Fig. 2E).

### Altering PAR metabolism leads to differential sperm gene nucleosomal association

To determine which genes were associated with abnormally retained histones in sperm, mononucleosomal DNA fractions were obtained using an established micrococcal endonuclease-digestion based chromatin fractionation method [31] and hybridized to promoter tiling array chips. Micrococcal endonuclease (MNase) digested sensitive mononucleosomal chromatin fractions were isolated and analyzed from separate sperm samples from individual sires (10 *Parg*(110)^−/−^, 4 PJ34-treated and 9 wild-type control males, Table 1 and Datasets S1, S2). Sensitivity to MNase cleavage was used as a surrogate measurement for histone association, exploiting the phenomenon that protaminated DNA is protected from cleavage, as reported by others [4], [31]. By subtraction of the background using sheared genomic DNA hybridized to separate chipsets as controls, unexpectedly more than 14,000 gene regulatory regions were found to contain at least some level of histones in control sperm samples (Table 1, Fig. 2F). There were individual differences between samples but multivariate principal component analyses using Partek software confirmed that MNase-sensitivity of sperm chromatin was more different between control and *Parg*(110)^−/−^ or PJ34-treated sperm samples than between samples within any single group (Suppl. Fig. S1 A, B). The large number of genes with MNase-sensitivity in the controls suggests that the residual presence of low, but detectable and statistically significant levels of nucleosomes in gene regulatory regions is normal for at least half of the ∼22,000 genes interrogated by the promoter tiling array. This finding is consistent with normal local residual sperm histone enrichment reported for many gene-rich chromosomal regions in mice [31].

**Table 1:** Genes associated with MNase-sensitivity.

Treatment/group	♂ [n]	Total genes [n]	MAT+ genes [n]	MAT− genes [n]
PargKO	10	11,777	-	-
Wildtype	9	14,060	-	-
PargA/Wt	1	4,877	2,320	3,027
PargB/Wt	1	4,854	2,301	3,022
PargC/Wt	1	4,922	2,148	2,957
All Parg/Wt	10	5,148	2,501	3,233
PJ34	4	14,787	-	-
PJ34A/Wt	1	6,063	3,700	3,089
PJ34B/Wt	1	7,740	4,039	4,773
PJ34C/Wt	1	5,945	2,567	4,077
All PJ34/Wt	4	7,839	4,582	4,791

*Parg*(110)^−/−^ (PargKO), PJ34, Wildtype controls: groups of sperm samples analyzed for gene histone association by tiling arrays with genomic “input” correction. Numbers of genes with significant histone binding in sperm are indicated. ♂ [n]: numbers of males. PargA/Wt, PJ34A/Wt, etc.: sperm samples of single males (♂, n = 1) analyzed for sperm histone association after pair-wise comparison to wild-type samples. Total genes [n]: number of sperm genes (differentially) associated with histones, indiscriminate whether sensitivity was increased (MAT+ genes [n]) or decreased (MAT− genes [n]). Please note that a number of genes were associated with both (MAT+ and MAT−) fractions. All Parg/Wt, All PJ34/Wt: comparison of all *Parg*(110)^−/−^ males (n = 10) with wild-type (n = 9) males or males treated with PJ34 (n = 4) with wild-type control males (n = 9).

The promoter tiling arrays used recognize promoter regions, as well as a number of exons in smaller genes and some 3′regions of select genes. These arrays are therefore blind to the vast majority of intergenic regions, so that calculation of relative enrichment of nucleosomes at promoters relative to the overall genome is limited by the design of the array chips. However, within the regions covered by the array, there was still a highly significant positive correlation of preferential histone association with promoter regions (Fig. S2A) and GC-rich DNA sequences (p<0.001, Wilcoxon test comparing MNase-enrichment between regions with high versus low histone association, Fig. S2B, C). These findings are consistent with known sperm histone patterns in human and mouse [4], [5], [7], [13], which our data sets reproduce to the extent possible given the dissimilarity of the techniques used. Similarly in line with these previous reports, sperm histone association was also inversely correlated with DNA methylation (Fig. S2C).

Pair-wise analyses of differential histone association in *Parg*(110)^−/−^ and PJ34-treated males compared to controls (Fig. 2F, G) indicated a significant overlap of differentially histone-associated genes between individual sperm samples (Fig. 2 F, G, H, red and blue circles). By comparison of data from all individual *Parg*(110)^−/−^ sperm samples with all wild-type data sets, 5,148 genes that were statistically significantly differentially histone-associated as measured by MNase-sensitivity (Fig. 2F) were identified in the overlaps (see also Datasets S1). In the same way we identified 7,839 genes in sperm from PJ34-treated males as common differentially histone-associated genes compared to controls (Fig. 2G, and Dataset S2). Furthermore, comparing the overlaps between the two models (Fig. 2H) identified 583 genes that were in both groups, corresponding to almost 3% of all mouse genes that was similarly affected by abnormal histone association after alternating PAR metabolism. This group was enriched in genes with ontologies broadly related to “nucleotide binding”, “GTPase activity”, “DNA binding”, “nucleic acid binding”, “cellular homeostasis”, “regulation of development” and “neural differentiation” as well as “metabolism” and “transcription” (false discovery rates (FDR): 0.005%–7%, see also Dataset S4). These genes likely reflect both a subset of the sperm genome that is subject to individual variation between males and true effects of altered PARP activity because pair-wise comparisons also showed differences between individual wild-type control samples, albeit to a lesser degree than between wild-type and experimental groups as indicated by different types of variance analyses (e.g., Fig. S1, and data not shown).

**Figure 3:**
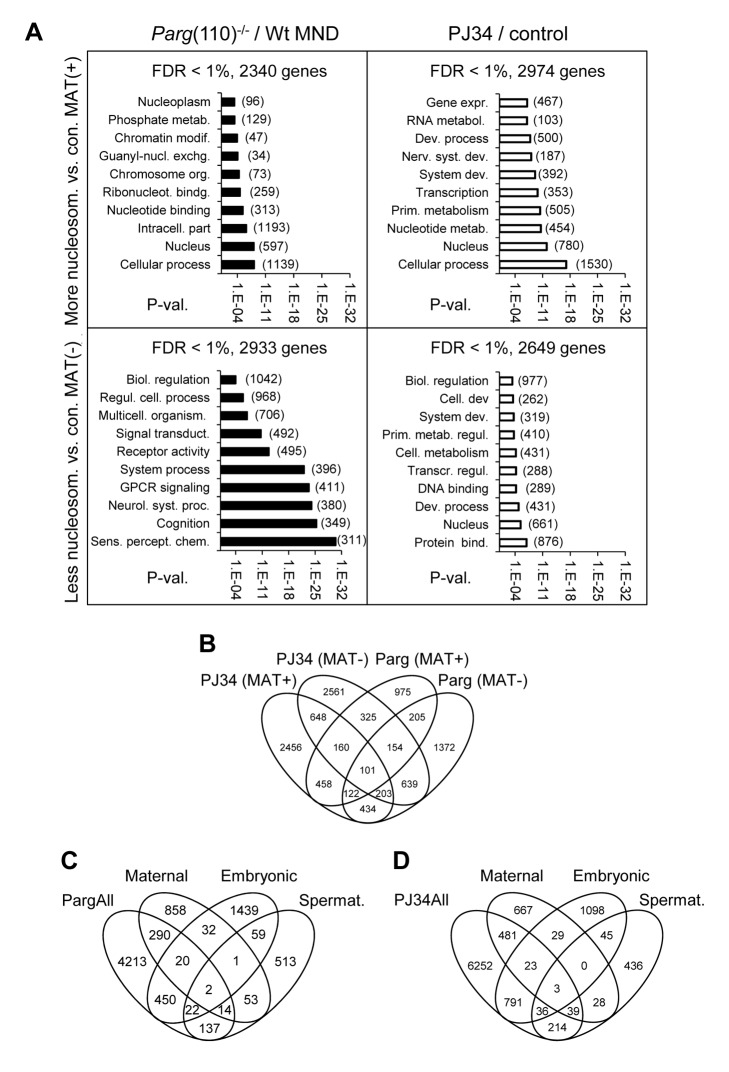
Perturbing PAR metabolism results in differential sperm histone association of gene loci with either excessive or reduced retention of nucleosomes. A) Functional GO-term enrichment of genes affected by elevated histone association (MAT(+)) or local failure to retain histones in regulatory gene sequences (MAT(−)) in sperm from *Parg*(110)^−/−^ (left panels) and PJ34-treated males (right hand panels). The y-axis shows GO terms and logarithmic scale indicates their p-values of GO-terms returned by DAVID. False discovery rates (FDR) are indicated above the graphs. The numbers of genes in a given GO-term are in parentheses. (B) Overlaps of relative histone enrichment or deficiency in *Parg*(110)^−/−^ or PJ34-treated mouse models compared to wild-type controls. (C, D) Comparison of genes that are differentially histone associated in *Parg*(110)^−/−^ or PJ34 sperm with known maternal transcripts or newly expressed embryonic transcripts or spermatogenesis-specific genes indicates the potential relevance of aberrant histone association on genes expressed in the 2-cell embryo (Embryo). Maternal: transcripts present in 1-cell embryos prior to the major wave of genome activation [36]. The genes in the maternal, embryonic and spermatogenic groups are listed in Dataset S4 (MS Excel).

Using Model-based Analyses of Tiling arrays (MAT) statistics [32], the genes associated with regions of relatively increased MNase-sensitivity (i.e., presumed histone enrichment in sperm derived from either knockout or PJ34-treated males compared to controls) were assigned positive MAT values (MAT(+)), whereas genes with histone depletion relative to the controls received negative MAT values (MAT(−)). Based on these analyses, both increased and decreased relative MNase-sensitivity of gene loci were observed. Finding that perturbing PAR metabolism not only leads to histone enrichment in genomic loci but also to the relative underrepresentation of nucleosomes in many other loci (Fig. 3A) was unexpected because the *Parg*(110)^−/−^ and PJ34-treated mice show a net excessive sperm histone retention. A possible explanation for this finding is that the net excessive retention of histones was mostly in repetitive, non-coding DNA domains and a stochastic, retention of histones in many genes in all sperm occurs in a given sample due to individual variation. In favor of this explanation is that fluorescent labeling of nucleosomal DNA extracted from sperm using MNase digestion, followed by *in situ* hybridization to wild-type sperm, yields preferential staining of the inner sperm chromocenter and the periphery of the nucleus [31], [33]. This finding indicates that only a minor fraction of sperm nucleosomes are retained on genes, whereas the majority of nucleosomes is bound to centromeric and telomeric heterochromatic regions. Similar results were obtained for PJ34-treated animals.

Gene ontology (GO) analyses with DAVID [34], [35] were used to ascertain whether the genes affected by differential sperm histone association coded for specific cellular functions, and the results indicated that a substantial number of gene groups was significantly affected by differential histone association. The functional gene groups were broadly similar to groups that are typically histone-associated ([13]) such as genes involved in cellular homeostasis, embryonic development and the stimulus of perception (Fig. 3A). Thus, differential sperm histone positioning due to aberrant PAR metabolism may occur genome-wide, likely being mostly stochastic but also in part patterned. The latter conclusion is suggested by the existence of groups of genes that were commonly differentially histone-associated in the PJ34-treated and *Parg*(110)^−/−^ sperm samples (overlaps in Fig. 3B). In summary, the data underscore the broad impact of perturbing PARP activity on sperm chromatin structure and histone association of genes in sperm.

Based on the known transcript profiles of oocytes, zygotes [36], and 2-cell embryos [37], the pool of differentially MNase-sensitive sperm gene promoters of *Parg*(110)^−/−^ and PJ34-treated males contained several hundred genes for which transcripts can be normally found in early stage embryos (Fig. 3C, D). This result raised the question whether expression of these genes is influenced in the embryo.

### Differential gene expression in 2-cell progeny from males with altered sperm chromatin composition

To identify genes that are differentially expressed in embryos, transcriptome profiling of individual 2-cell embryos fathered by *Parg*(110)^−/−^, PJ34-treated or appropriate control males was performed using microarrays, as well as deep sequencing (see Datasets S1, S2, S3). We analyzed individual embryos because they are the product of a single sperm, which enabled us to compare directly changes in histone composition of sperm in a given male with expression of these genes in his progeny.

**Figure 4:**
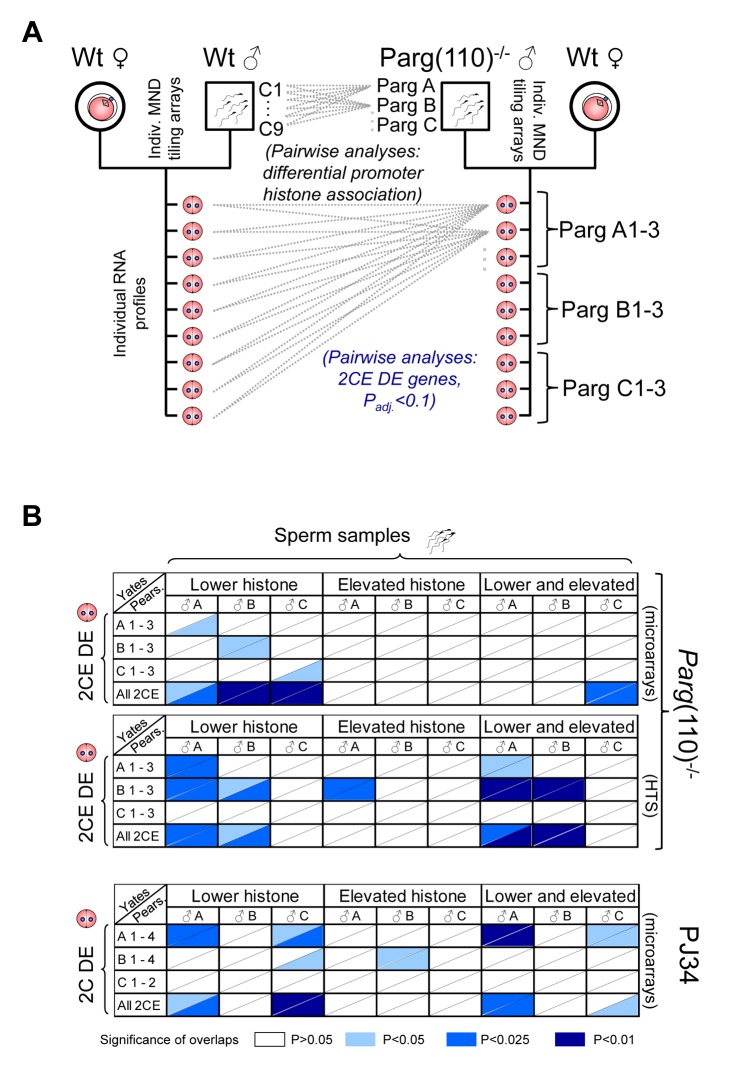
Differential sperm histone association of genes in *Parg*(110)^−/−^ and PJ34-treated males is significantly associated with differential expression of these genes in offspring 2-cell embryos. (A) Outline of the comparison procedure, shown here only for the *Parg*(110) gene disrupted mouse model. A similar regimen was used for the PJ34-treated males and their offspring. Differential histone association of genes in sperm from individual males (PargA, PargB, PargC) was determined by pair-wise comparison with all individual wild-type data sets obtained from 9 individually analyzed control males. Differential gene expression was determined by pair-wise comparison of individual offspring from the *Parg*(110)^−/−^ males (PargA1-PargC3) with all 9 individual wild-type 2-cell embryo data sets by either microarrays or next generation sequencing. Differential gene expression was determined using ANOVA analyses and adjusted P-value calculation (with P_adj_<0.1 considered to be significant). (B) Pearson (uncorrected) and Yates (corrected) Chi-squared tests were used to determine the significance of overlaps of the lists of genes that were differentially histone associated in sperm samples of the sires (“Sperm samples”) compared to controls (Parg A–C and PJ34A–C), with the lists of genes that were differentially expressed in at least one of the 3 or 4 offspring 2-cell embryos from these sires (“2CE DE”, *Parg*(110)^−/−^: A 1–3, B 1–3, C 1–3, and for PJ34: A1–4, B1–4, C1–2). A genetic background of 19,472 genes interrogated by the microarrays and 20,018 genes interrogated by the tiling arrays and sequencing platforms was used for the calculations. Ranges of P-values resulting from Yates or Pearson are indicated in different shades of blue if P≤0.05, i.e., the overlaps were significant (see color legend in figure). The P-value denotes the confidence with which the null-hypothesis can be dismissed that the overlap between the list of genes with abnormal histone association in the sire with the list of genes that are DE in the offspring could be predicted by statistical probability, i.e. coincidence. Upper two panels: *Parg*(110)^−/−^ group of fathers and offspring embryos, microarray expression analyses with either Yates' chi-squared test (upper triangular portion of each cell) or Pearson's (lower triangular portion of cells). HTS: *Parg*(110) group, high throughput sequencing of 2CE gene expression. Lower panel: overlaps of PJ34-treated group of fathers (sperm) and offspring embryos (2-cell embryo differential gene expression, “2CE DE”). Note that mainly overlaps between genes with lower histone retention in sperm and differentially expressed genes in offspring embryos are significant with P≤0.05 (left 1/3 portion of each panel).

Because large populations of sperm and not individual sperm were analyzed here, the expectation was that identifying a particular gene as differentially histone-associated simply reflects that it is affected in a certain fraction of the ∼10^6^ sperm analyzed per single male, i.e. MNase digest/tiling array. If the abnormally placed histones in that gene locus have the potential to change gene expression later in the embryo, similarly only a certain percentage of embryos will exhibit altered expression of the gene. Given the differences between individual males and the natural variability of spermatozoa, as reflected by the broad spectrum of sperm fluorescence intensities measured in the CMA3 assays (Fig. 2A–D), a low signal-to-noise ratio was expected, making statistical analyses of pooled embryos impractical. Accordingly, the experimental design was such that individual embryos, which are each the product of a single sperm, were used for genome-wide, transcriptome analyses instead of pools of embryos (Fig. 4A). Microarray expression data sets were subjected to a number of different variance analyses between individual wild-type control, *Parg*(110)^−/+^ and PJ34 embryos (e.g. Fig. S3, and data not shown). Differences in the average coefficient of variation (Cv) between controls and experimental groups were statistically significant (i.e., 4.29% in the *Parg*(110)^−/+^, 4.77% in the wild-type control and 4.59% in the PJ34 group) due to the large number of genes interrogated, but overall small. Genes identified as highly variable in the controls (Cv>5%) were not excluded from the overall analyses because their contribution to the pool of genes identified as differentially expressed was only less than 4% and variation was similar between control and experimental groups. Overall differences in gene expression profiles were small between control, *Parg*(110)^−/+^ and PJ34-treated samples.

The total number of genes differentially expressed in the 2-cell embryos (2CEDE) varied depending on the individual embryo and ranged from 88–407 genes (average = 138) in the *Parg*(110)^−/−^ offspring to 35–401 genes (average = 110) in the PJ34 model (total numbers listed in Table 2). These numbers correspond to only 0.2–2% of all interrogated genes, illustrating that overall differences in the gene expression profiles of embryos were small. Moreover, with respect to our central hypothesis this finding suggests that only a minority of differential sperm histone association events can be expected to have the ability to change gene expression in the 2-cell embryo.

**Table 2:** Genes with differential expression in 2-cell embryos.

Exp. Group	2CE DE genes [n]	DE ratio>1 [n (%)]	DE ratio<1 [n (%)]
Parg(110)^−/−^(arrays)	1241	972(78.3)	269 (21.7)
Parg(110)^−/−^ (NGS)	370	337 (91.1)	33 (8.9)
PJ34 inj. (arrays)	1095	869 (79.4)	225 (20.6)

Separating differentially expressed genes in 2-cell embryos (2CEDE) into up-regulated (ratio>1) and down-regulated genes (ratio<1) reveals a strong bias of differentially-expressed genes towards up-regulation or illegitimate activation of genes across all experimental groups/platforms used. The numbers in brackets indicate percentage of genes in a category, e.g., 75.4% of all genes detected in the microarrays of *Parg*(110)^−/−^ 2CE DE were up-regulated and 24.6% were down-regulated.

Microarray data validation was performed by next-generation sequencing of the same individual 2-cell embryos derived from either wild-type or *Parg*(110)^−/−^ sperm that were also subjected to microarray analyses (Dataset S3). The overall similarity of the data sets validated integrity of the microarray data (Suppl. Fig. S4). Because the high-throughput sequencing (HTS) data were generated using material that had previously undergone a whole-transcriptome amplification step, we utilized the microarray results as the primary data sets for further analyses. The PJ34 microarray results were validated by quantitative PCR analyses of nine differentially expressed genes and four control genes (Fig. S5). In summary, individual offspring of *Parg*(110)^−/−^ or PJ34-treated males showed highly significant differential expression of genes in the 2-cell embryo stage compared to embryos of the control animals.

### Sperm chromatin structure influences gene expression in the embryo

Unless genes are uniformly differentially expressed in all of the individual 2-cell embryos derived from *Parg*(110)^−/−^ or PJ34-treated fathers compared to the controls without any exception, they will be in part also detected as highly variable in the experimental groups (with a Cv>5%). Such variation is expected given the heterogeneous nature of individual sperm populations from which the embryos were derived (Fig. 2B, D). We reasoned that differential expression of these genes would only be of significance if they are also found to be differentially histone-associated in sperm samples from which they are derived. This connection was determined by comparing sets of differentially expressed genes in 2-cell embryos with genes of differential nucleosomal organization (tiling arrays of MNase fractions) in sperm to ascertain whether a positive correlation existed. We therefore performed pair-wise comparisons between the genes identified as differentially histone-associated in sperm of a particular male, with the gene lists describing genes that are differentially expressed in his individual offspring compared to the pooled wild type control embryos (2-cell embryo differentially expressed; 2CEDE) (Level 1 list) and all offspring from the other males (Fig. 4A and B), using Yates' corrected or Pearson's Chi-squared tests. These tests compare the prediction with which an overlap of two populations in a limited space (all 19,472 genes interrogated) occurs merely by chance (null hypothesis) with the number of actually observed events of overlap between the two groups. Overlaps of these lists of genes were deemed significant if the resulting P-value was ≤0.05 and the mathematically expected random overlap was indeed smaller than the observed overlap. Due to the small numbers of differentially expressed genes and the mathematical nature of contingency table calculations, overlaps of individual single embryos with paternal sperm samples (large number of genes) were often not feasible. Therefore, all differentially expressed genes identified in at least one of the offspring belonging to a given male were listed together to generate a “family” list of genes (Level 2 list, the results are shown in Fig. 4B). Finally, all differentially-expressed genes of all embryos within the knock out, PJ34-treated or control groups were pooled to generate a Level 3 list (“genotype” or “treatment”).

Strikingly, most overlaps between the lists of genes that were differentially expressed in the *Parg*(110)^−/+^ embryos and the list of genes that had lower histone retention in sperm compared to the wild-type controls were significant at Level 2 (family) or Level 3 (genotype) (highlighted boxes in Fig, 4B, for exact P-values see Fig. S9). These genes were identified by the MAT analyses as the “MAT(−) group” of genes which have abnormally reduced MNase sensitivity and therefore lower histone association compared to the wild-type. In contrast, overlaps between the MAT(+) group (representing increased histone retention in the experimental groups of perturbed PAR metabolism) and the differentially expressed gene groups tended to be not significant. Taken together, the observed association of abnormal histone retention of genes with their differential expression in the preimplantation embryo (subsequently termed “2CEDE/MND” genes) was determined to be significant using the described mathematical tests. These results support the hypothesis that an inappropriate degree of histone association of a given gene in sperm affects expression of that gene in the early embryo.

### Variegated sperm histone retention correlates with altered expression of ribosomal protein genes and genes important for neural development and may modulate pluripotency gene expression

**Figure 5:**
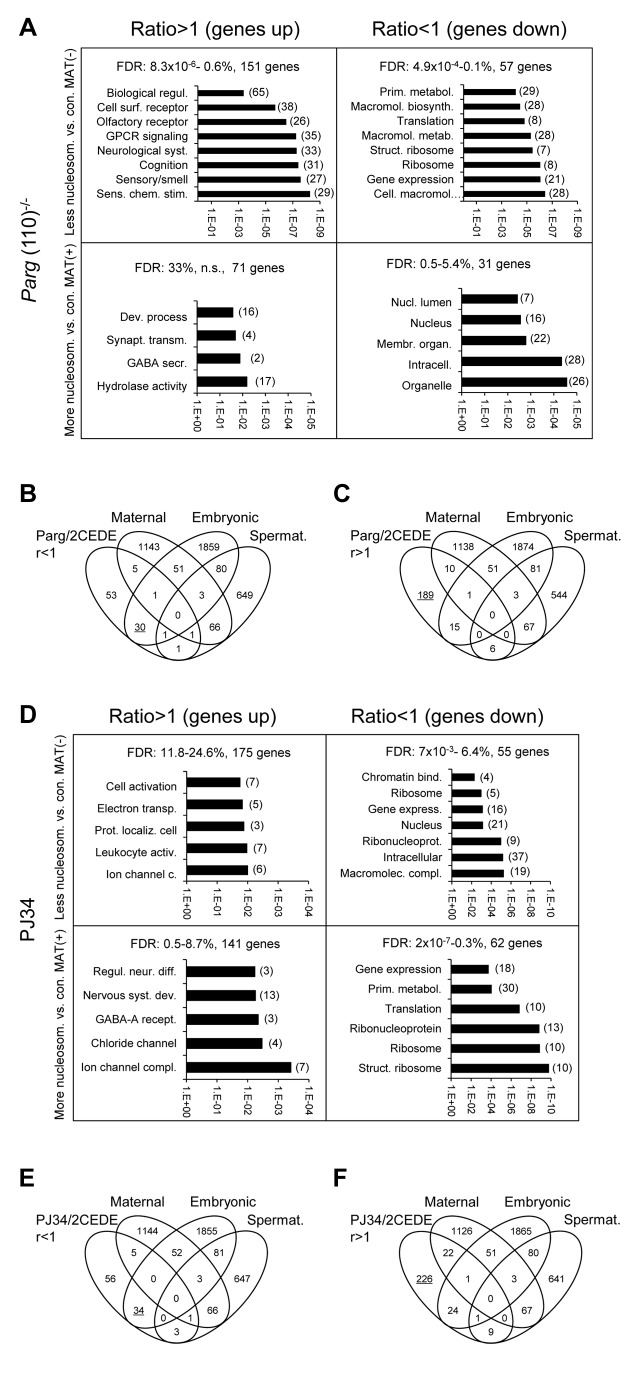
Genes that are affected by altered sperm histone association and differential gene expression in 2-cell embryo progeny in the *Parg*(110)^−/−^ (A, B, C) and PJ34-treated (D, E, F) mouse models share some common functional relevance. Analyses of functional ontology of genes that are both affected by elevated (MAT(+)) or lower (MAT(−)) differential histone association in sperm and differential expression in resulting embryos (2CEDE/MND) were performed in the *Parg*(110)^−/−^ (A) or PJ34 (D) model system. Genes are listed according to up- (ratio>1) or down (ratio<1) regulation compared to controls in offspring from *Parg*(110)^−/−^ or PJ34 treated males and p-values of the GO terms (DAVID) associated with the corresponding group of genes are given on the logarithmic x-axis. The number of genes in each GO functional category is in parentheses. FDR: false discovery rates. (B, C, E, F) Comparing lists of genes that were either down-regulated (B, E) or up-regulated (C, F) in offspring of *Parg*(110)^−/−^ or PJ34 treated males compared to controls (taken from (A) or (D)) to lists of genes of known embryonic [37], or maternal [36], origin demonstrated that maternal transcripts left over from the zygote were largely unchanged. Down-regulated transcripts (ratio<1) in 2-cell embryos of fathers with perturbed PAR metabolism were mostly of embryonic, but not maternal origin (B, E). Up-regulated genes are those that are not normally expressed during this phase of embryo development (C, F, underlined). These genes appear precociously expressed here and are normally only active after the blastocyst stage of development.

Functional gene ontology group (“GO-term”) analyses of 2CEDE/MND genes using DAVID (Fig. 5A, D) yielded similar GO terms for both the *Parg*(110)^−/−^ and PJ34-treated mouse models. GO-terms for the group of genes that were aberrantly up-regulated in embryos were moderately significant, suggesting that their proper nucleosomal organization in the sperm normally has a silencing function in the early embryo. These genes, which are only loosely grouped functionally, can be described as genes typically expressed only later in embryonic development, such as olfactory receptors, ion channels and other genes involved in neuronal development (Fig. 5A, D). In contrast, ribosomal protein genes and other genes involved in gene expression and metabolism were found to be expressed at reduced levels in embryos derived from *Parg*(110)^−/−^ and PJ34-treated sires with high confidence (Fig. 5A, D). In summary, based on these observations, aberrant histone retention due to altered PAR metabolism was associated with a general up-regulation of developmental genes in 2-cell embryos, and down-regulation of metabolic genes involved in cellular homeostasis.

Notable exceptions of this general observation were, for instance, genes involved in epigenetic chromatin modulation such as *Kdm4c*, *Hells/Lsh*, *No66* or *Setdb2* (Fig. S5). These genes were also in the group of genes affected by both abnormal histone positioning and differential expression in offspring of PJ34 treated animals. The pluripotency genes *Pou5f1/Oct4*, *Myc*, *Sox2* and *Klf4* were among the genes with the highest elevation of histone retention in sperm from PJ34 treated males compared to the wild-type but only *Pou5f1* expression was also differentially (down-) regulated in embryos, whereas the other genes were not yet significantly expressed in any of the embryos (Fig. S6).

### Sperm histone placement affects embryo genome activation but not maternal mRNAs

RNA transcripts found in late 2-cell embryos are either newly synthesized by the embryo undergoing genome activation or are maternal transcripts not yet degraded. To determine the origin of differential transcript frequencies in the 2-cell embryos fathered by *Parg*(110)^−/−^ or PJ34-treated males, the 2CEDE/MND genes were compared to the known expression profiles of oocytes, 1-cell, 2-cell, 8-cell and blastocyst stage embryos. For these analyses, a “Maternal” group gene list was assembled from transcripts predominantly found in the oocyte and the 1-cell embryo prior to the major embryonic genome activation in the 2-cell embryo [36]. The “Embryo” group comprised transcripts from genes with α-amanitin-sensitive expression in the 2-cell embryo, i.e., zygotically-expressed transcripts [37]. Comparisons demonstrated that a significant percentage of the aberrantly down-regulated genes (2CEDE/MND) was of embryonic origin (33% in the *Parg*(110)^−/−^ and 34% in the PJ34-fathered 2-cell embryos, P<0.0001, Chi-square test with Yates's correction, Fig. 5B, E, Table 2). In contrast, only 4–7% of this group of genes represented transcripts that could also have been of maternal origin (Fig. 5B, E). Interestingly, the differentially expressed genes (2CEDE/MND) that had aberrantly high transcript frequencies in the embryos (ratio>1) were not limited to the previously described group of α-amanitin-sensitive embryonic genes (Fig. 5B, E) and were not significantly related to the “Maternal” group. The data suggest that at least some of these up-regulated transcripts represent genes that are normally only expressed later during development after the blastocyst stage but were now precociously expressed in the 2-cell embryo. Interestingly, all differential gene expression analyses show a similar tendency towards higher expression of genes or new expression of genes that are not normally expressed in the 2-cell embryo (Table 2) rather than decreased expression of genes, which represent the minority.

Taken together, these data indicate that embryonic gene expression in the 2-cell embryos obtained from *Parg*(110)^−/−^ and PJ34-treated sires highly correlated with differential sperm histone retention, but not with the metabolism of maternal transcripts. In these analyses, we also included a comparison with genes specifically expressed in spermatogenesis but no significant overlaps were detected (see also Fig. 3).

### Perturbing PAR metabolism affects a common subset of genes in both mouse models

**Figure 6:**
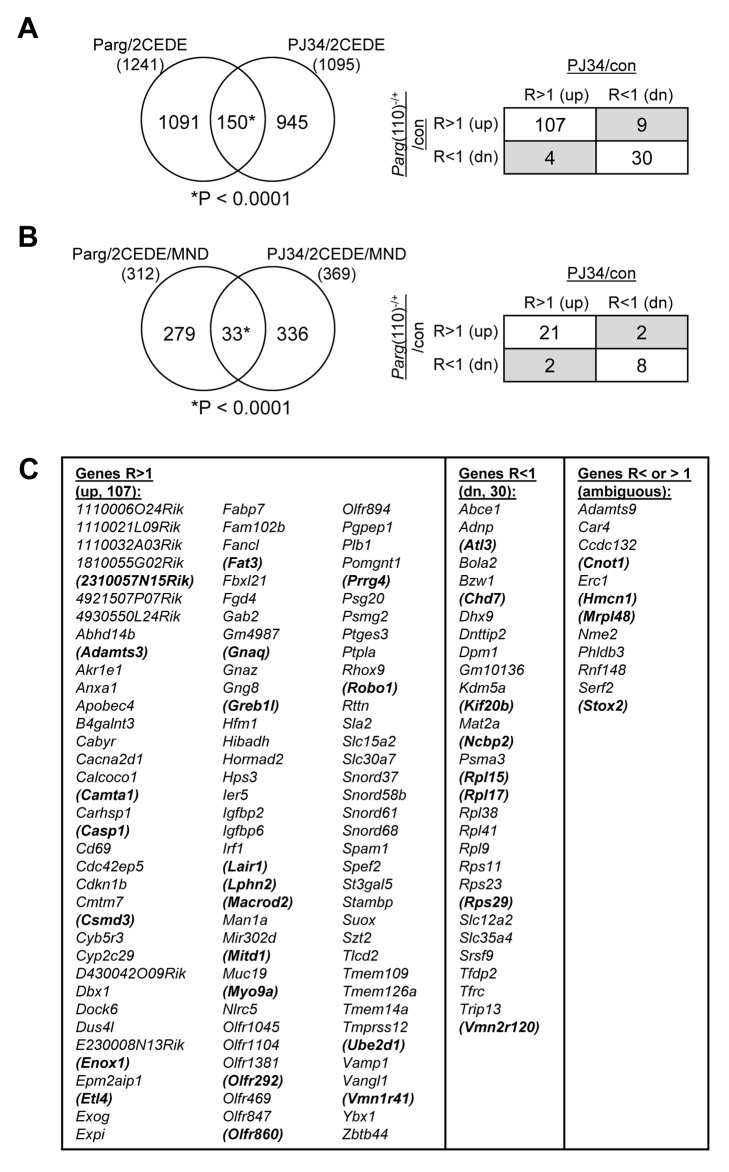
Shared differential gene expression in offspring from males of the two mouse models with perturbed PAR metabolism (*Parg*(110)^−/−^, PJ34 treated). (A) The overlap of 150 differentially expressed 2-cell embryo genes (2CEDE) from *Parg*(110)^−/−^ and PJ34 treated males is highly significant (Yates' and Pearson's Chi-squared tests using a genetic background of 19,472 genes, P<0.0001 in both analyses, the null hypothesis would be 70 genes in the overlap). Of the 150 genes commonly differentially expressed in embryos of the two mouse models of reduced PAR metabolism, 107 are commonly expressed at higher levels and 30 are commonly down-regulated (right panel). The shaded fields indicate genes with variable expression; these also have high coefficients of variation (Cv>5%) in the variance analyses. (B) There is also a significant overlap of 33 genes that were both differentially expressed in individual embryos and differentially histone associated in the corresponding sperm sample (2CEDE/MND genes) between the two models (*Parg*(110)^−/−^ and PJ34-treated fathers (P<0.0001, the null hypothesis would have been 4.8 genes in the overlap)). The relationships of differential expression of these genes (ratio>1: R>1 or ratio<1: R<1) are again very similar for the genes in the overlap (box panel to the right). (C) Identity of the genes in the overlaps shown in (A). The 33 genes in the overlap shown in (B) are bold and in brackets. Variably (ambiguously) expressed genes are listed in column 5.

Comparing differential gene expression in 2-cell offspring from *Parg*(110)^−/−^ and PJ34-treated males showed that 150 genes were commonly differentially expressed. Chi-squared analyses confirm the significance of this overlap of 12% (*Parg*(110)^−/+^) and 13.7% (PJ34) of all differentially expressed genes in these embryos (Fig. 6A) with very high confidence. Importantly, differential expression of the majority of these genes (137) follows similar patterns (up- or down-regulated) when PAR metabolism is perturbed in fathers using both models (Fig. 6A, right panel and lists in Fig. 6C). The remaining 12 genes (gray fields in Fig. 6A, right panel) belong to the group of genes with highly variable expression levels with a coefficient of variation that was higher than 5% previously identified by our variance analyses. In addition, the PJ34 and the *Parg*(110)^−/−^ gene disrupted mouse models shared a small, but significant, overlap of 33 genes that were both, differentially histone associated in sperm and differentially expressed in embryos (2CEDE/MND) (P<0.0001, Yates corrected chi-squared test, Fig. 6B and listed in Fig. 6C, genes in brackets). Within this group, these genes again had similar modes of differential expression (up with a ratio of r>1, or down with r<1), and similar tendencies of differential sperm histone association (MAT(+) or MAT(−)) in the two models (Fig. 6B, right panel). The overlap represents a common group of 33 genes affected by altering PAR metabolism and hence sperm histone association, in the two models. Functional ontology analyses of all of these groups of genes in the overlaps between the Parg(110)^−/−^ and the PJ34-treated models (using 150, 137, 107, 33, or 29 genes, see Figs. 6A, B) always indicate an enrichment of ribosomal protein genes (9 genes that are down-regulated). The highest significance of this GO term was returned for analysis of the 30 commonly down-regulated genes (Fig. 6C, fourth column, GO:0003735 ∼structural constituent of ribosome, p = 4.5×10^−9^, FDR = 5×10^−6^). The identity of commonly differentially expressed genes is shown in Fig. 6C and in Dataset S4 (MS Excel).

## Discussion

Using gene expression and DNA tiling arrays, high-throughput sequencing and microarrays, i.e., employing three different platforms interrogating two different mouse models, our study supports the hypothesis that mammalian sperm chromatin carries epigenetic information that persists throughout remodeling of the paternal pronucleus in the zygote and can influence gene expression during the course of genome activation.

Whether retained sperm histones influence gene expression in embryos has been addressed using a variety of techniques, including chromatin immunoprecipitation with histone and histone modification-specific antibodies in combination with DNA deep sequencing to map gene histone association in human and murine sperm at very high resolution [4], [5], [7], [13], [31]. Together with proteomic and in silico approaches, these investigations have yielded significant progress in our understanding of postmeiotic reprogramming of the male genome in mammals (reviewed e.g. in [38]) and important principles underlying the selective retention of histones during spermiogenesis are beginning to emerge [13]. Most of these mechanistic insights were gained by comparison of well characterized patterns of known gene expression in the embryo and in spermatogenic cells with the patterning of sperm histone retention. The present study uses a different approach by changing histone association in sperm followed by gene expression analysis in the preimplantation embryo resulting from such sperm and thereby advances our understanding how chromatin-based epigenetic inheritance is modulated.

In normal human sperm, nucleosomes, possibly modified, are retained preferentially at regulatory sequences and around transcriptional start sites [5]. Because sperm histones likely persist on the paternal genome post-fertilization, a consequence is that post-translational modifications of retained histones, e.g., H3K27-, H3K9- or H3K4 trimethylation, may be able to influence the level of expression of the affected genes later in the embryo. Because there are waves of DNA demethylation and remethylation of the paternal chromosome complement in the early embryo, the presence or absence of such modified histones could also have an impact on DNA methylation. The histones retained in sperm are also associated with GC-rich DNA sites frequently found in promoter regions and in transcriptional start sites of housekeeping genes [5], [7], [39]. These studies also reported a correlation between promoter regions with sperm histone retention in human and GC-rich sequences that remain unmethylated in ES cells, suggesting that retained sperm histones prevent DNA methylation of GC-rich sites they occupy in the early embryo.

**Figure 7:**
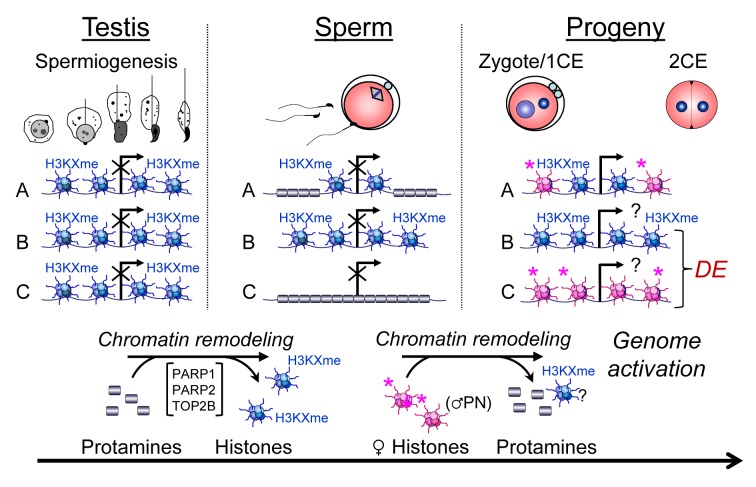
Chromatin remodeling events in spermiogenesis affect sperm histone-dependent regulation of gene expression during embryonic genome activation (working model). Chromatin remodeling during spermiogenesis (left panel) leads to the exchange of nucleosomes (blue) with their specific paternal tail modifications by protamines that normally leads to regulated retention of histones in certain domains of a given gene locus (A). Modulation of this remodeling process, for instance by altering PAR metabolism, results in either insufficient exchange of histones (B) or excessive remodeling causing more intense depletion of histones from that locus (C). As a result, histone association of this locus can be variable in sperm (middle panel). After fertilization, the paternal chromatin becomes rapidly remodeled, again with the exchange of protamines, but presumably not paternal histones, for maternally provided histones (pink) with maternal tail modifications that are mostly activating or nondescript in nature (right hand panel). As a result, the ratio of maternal and paternal histones can vary at the time point of genome activation, leading to continued differential epigenetic remodeling of the locus and ensuing differential expression (DE) in the early embryo.

We anticipated identifying subsets of gene regulatory regions that would be characterized by either a complete absence or presence of sperm histones, but found that this expectation was not met. Rather, we find that a very large number of genes (∼14,000, similar to findings by others [39]), if not all genes (considering our subtraction of genomic DNA signals that may have introduced a sensitivity threshold below which genes would not have been detected) carry histones in sperm, albeit to varying degrees (also see Fig. S7). This observation suggests that a large proportion of the genes in sperm are normally marked by histones that act as bookmarks to guide their expression in the early embryo. In this scenario, histones could also preserve the epigenetic profile of the paternal genome by ensuring correct demethylation and remethylation of gene promoters during embryonic reprogramming steps because DNA methylation and histone modifications are intimately related. The methylomes of human sperm and ES cells exhibit gene-associated hypomethylation in more than 70% of all annotated genes, lending support to the view that promoter regions are generally identified and bookmarked in sperm [40]. Interestingly, the presence of H3K4me3 at promoters is often accompanied by DNA hypomethylation [7], [40]. H3K4me3 is an activating histone mark thought to inhibit locally DNA methylation at promoter sites it occupies and therefore may protect genes from DNA methylation during reprogramming steps later in development, i.e., post-fertilization. In sperm and spermatids, H3K4 methylation often occurs together with H3K27me3, a mark involved in polycomb-mediated gene repression but does not promote DNA methylation. A large fraction of genes marked with both histone modifications in sperm remain suppressed during preimplantation development, e.g., some genes encoding pluripotency factors [13]. Consequently, the loss of histones in a given locus during spermatid maturation could result in loss of the epigenetic information in that locus (Fig. 7), possibly leading to the formation of an epimutation, e.g., by excessive or insufficient DNA methylation. Conversely, failure to evict modified histones from promoters leading to abnormally elevated histone retention in a given locus could alter reprogramming and DNA methylation and thereby regulate gene expression by promoting polycomb repression.

An interesting observation is that the pluripotency factors *Pou5f1/Oct4*, *Myc*, *Sox2* and *Klf4* are among the most excessively histone-associated genes compared to the wild-type in two of the three PJ34-treated sperm samples. These genes also have a relatively high baseline of histone association in wild-type sperm [1]. However, only *Pou5f1*, which is the only one of the four genes normally expressed in 2-cell embryos, is also differentially expressed at a lower level in a PJ34C offspring (Fig. S6B). This result suggests that PARP activity has an impact on histone eviction at promoter regions of pluripotency genes, and paternal inhibition of the pathway may therefore modulate expression of these essential genes in later stages of offspring development, e.g., the blastocyst, when these genes are normally expressed. Further investigations will confirm whether this is the case.

More than 5,000 promoters in human sperm lack hypomethylated regions and have low histone retention [40]. These genes are highly enriched in G-protein coupled receptors and genes involved in neurological functions. Such genes are normally associated with highly specialized cell types and our data suggest that these genes may be more sensitive to alteration of histone association in sperm and subsequent differential expression in the 2-cell embryo when paternal PAR metabolism is perturbed (Fig. 5A, D). Olfactory receptor genes represent an example of such genes; they are marked by a low GC content and a low degree of histone association (Fig. S7), especially over their coding regions, which appears to be similar in ribosomal protein genes (Fig. S6A). For many of these genes we observed precocious embryonic expression of individual genes linked with abnormal depletion of histones (i.e., decreased MNase sensitivity) from the corresponding locus in sperm of *Parg*(110)^−/−^, and to some extent in PJ34-treated males (Fig. 5). Olfactory gene clusters were consistently characterized by an overall low baseline abundance of sperm histones except in the promoter and 3′ untranslated regions of the individual genes in our analyses (Fig. S7). Olfactory genes represent the largest group of genes in both humans (∼850 genes and pseudogenes [41]) and mice (1,200–1,500 genes [42]), which may have contributed to their identification as differentially-expressed genes in our investigations. Expression of the vast majority of these genes is strictly regulated in neuronal development and only a single receptor gene is normally expressed per single neuron [43], [44]. Overall, regulation of olfactory receptor expression is not well understood [42], but our data suggest that the binding of a small number of histones in key positions could contribute to silencing of these genes from the paternal genome immediately after fertilization; if these histones are lost in sperm, expression of these genes is then no longer suppressed in the early embryo. Ribosomal protein genes also normally appear relatively devoid of nucleosomes over their exons and promoters despite relatively high GC contents in these regions, and abnormal retention of histones in sperm of sires is associated with differential (down-) regulation of their expression in the embryo (Figs. 5, S6A).

Deficient sperm histone retention, i.e., the loss of histones by complete replacement of all histones with protamines, could occur with a certain stochastic probability in a given locus but the sheer size of the olfactory receptor and ribosomal protein gene families likely allowed detection of these groups in functional ontology analyses despite the limited number of embryos interrogated. However, the large number of 2CEDE/MND genes in progeny of *Parg*(110)^−/−^ and PJ34-treated males (Fig. 5) that are not enriched in these two functional (olfactory receptor and ribosomal protein) groups suggests that differential gene expression, concomitant with differential histone retention in sperm, also occurs to a large extent in other genes. These genes may be regulated similarly by retained sperm histones, but with a probability that would require larger sample sizes to determine the frequencies of events in these loci. Overall, this interpretation is consistent with the proposal that sperm histone-dependent regulation of embryonic gene expression is a basic biological mechanism that increases phenotypic variation [5] due to the variation of sperm histone retention and that excessively retained sperm histones found in patients with subfertility do not follow any discernible common patterns in their positioning in the genome and appear randomly [7].

As noted above, the strength of the experimental design is that sires and individual embryos were analyzed in pairs, which minimizes effects of imperfect penetrance of the sperm chromatin phenotype and variation between individual males. The limited amount of material that can be obtained from a single male mouse sperm sample, however, currently precludes using concomitant chromatin immunoprecipitation (ChIP) analyses to identify the types of histone marks found at promoter regions of the genes that are subsequently differentially expressed in 2-cell embryos. In addition, single 2-cell embryos are not yet readily amenable to DNA methylation analyses at specific sites so that the possibility of a direct link between histone association in sperm, differential gene expression and DNA methylation status in the 2-cell embryo still remains to be investigated.

The PARP inhibitor experiments generated results remarkably similar to the genetic *Parg*(110)^−/−^ model regarding the abnormal positioning of histones on gene promoter regions. Differential gene expression of embryos generated in the two model systems was similar in 150 genes with similar tendencies of higher or lower differential expression. The aberrant sperm nucleosome association is again highly significantly correlated with differential expression of genes in 2-cell embryos compared to control embryos (Fig. 6A, B). In addition, 33 genes are commonly present in the 2CEDE/MND groups of the two mouse models (Fig. 6B). The result that analyses in offspring from two different models of perturbed PAR metabolism produce in part similar outcomes suggests that the impact of PAR on sperm chromatin structure and histone association of genes in sperm is perhaps not predominantly stochastic in nature.

The finding that manipulation of PARP activity during spermiogenesis alters histone retention in sperm is consistent with the multiple functions of PARP1 and PARP2 as post-translational modifiers involved in the local decondensation of closed chromatin structures necessary for execution of DNA repair, transcription, and development [45], [46]. PARP1 and PARP2 become enzymatically activated by DNA strand breaks to synthesize PAR by cleavage of NAD^+^ into ADP-ribose and nicotinamide. PARP activity provides chromatin access and thus facilitates the histone-protamine exchange in elongating spermatids where DNA strand breaks are formed by the DNA relaxing activity of topoisomerase IIb (TOP2B) [27], [47]–[49]. PAR's high electronegative charge enables it to compete with DNA for binding of core histones, histone H1, and other proteins associated with DNA and thus remove these histones from the DNA. Auto-modification of PARP1 and PARP2 with PAR inhibits PARP1/2, and removes them from chromatin. Degradation of PAR into monomeric ADP-ribose by PAR glycohydrolase (PARG) is necessary to restore PARP activity, and rapid cycles of PAR formation and degradation account for transient, local chromatin decondensation events. Therefore, reducing the PARP regenerating activity of PARG in the hypomorphic *Parg*(110)^−/−^ mouse has a similar inhibitory effect on PARP activity as using a PARP inhibitor such as PJ34. In both cases, PARP inhibition interferes with chromatin opening and thus with the correct remodeling of spermatid chromatin, but the exact mechanism remains to be elucidated in future investigations.

Sperm gene histone retention on promotor regions depends, in part, on previous transcriptional activity in round spermatids and the concomitant exchange of canonical H3 variants for histone H3.3 [13]. Intriguingly, both PARP1 and PARG also play important roles in transcriptional regulation [50]. In addition, PARG also regulates PARP mediated PARylation of the histone H3K9 demethylase KDM4D/JMJD2D necessary for retinoic acid receptor (RAR)-dependent gene expression [51]. In the absence of PARG, KDM4D is excessively PARylated and unable to remove H3K9 methyl groups that block RAR-dependent gene transcription. *Parg*(110)^−/−^ mice have a residual PARG activity of ∼25% [52]. It is plausible that abnormal regulation of KDM4D and likely other histone demethylases such as KDM5D [53] contribute to the observed abnormal spermatid chromatin regulation and composition. The PARylation of histone demethylase KDM5D regulates genome-wide methylation of H3K4 and the inhibition of PARP activity by an inhibitor such as PJ34 as used in our investigations, or disruption of the *Parg* gene in *Parg*(110)^−/−^ males could therefore have an impact on spermatid gene expression and hence chromatin composition. Moreover, it is possible that altered histone methylation by perturbed PARP-dependent regulation of histone demethylases has a direct impact on nucleosome eviction during spermiogenesis and would be consistent with our observation of elevated histone retention in sperm of our mouse models (Fig. 2E and [27], [29]).

PARylation is also a crucial regulator of the insulator protein CCCTC-binding factor (CTCF) and its ability to form chromatin loops [54], [55]. CTCF-binding sites are highly enriched in MNase-sensitive sperm DNA fractions in both, humans [4] and mice [31]. Future studies addressing how the sites of histone retention in sperm are determined are clearly required.

PAR metabolism is emerging as a pathway monitoring environmental factors such as diet and chemical exposure of various kinds [56]. Examples of an epigenetic inheritance of metabolic disorders through the male germline have been described but the underlying mechanisms are not well understood [57], [58]. It is tempting to speculate that such heritable epigenetic memory of the paternal metabolic state could occur in the form of differential sperm histone retention by alteration of metabolic pathways including PAR metabolism, and likely several others, through an impact on chromatin remodeling in spermiogenesis.

When taken together, our genome-wide investigations demonstrate that perturbing sperm chromatin structure as a consequence of abnormal retention of histones during spermiogenesis leads to abnormal gene expression profiles in early preimplantation embryos (Fig. 7). Although the observed changes in gene expression are not detrimental to embryo survival, as most embryos can develop to term (data not shown), the data nevertheless provide, to our knowledge, the first experimental evidence of the postulated basic biological principle that association of gene promoter regions with histones in sperm regulates the expression of those genes after fertilization in the resulting embryo. The assumption is that the perturbations observed in gene expression are derived from transcription of the paternal genome. Although we cannot at present state that such is the case, ongoing RNA sequencing experiments using inter-strain crosses should permit identification and quantification of gene expression levels derived from either the maternal and paternal genomes by single nucleotide polymorphisms. Finally, our findings support the view that epigenetic information contained in the sperm nucleus can survive the dramatic chromatin remodeling process that occurs in the male pronucleus. Our findings collectively support the view that nucleosomal association of a sperm gene locus is informative to gene expression in the preimplantation embryo, as previously proposed ([1], [2], [5], [6], [19], [24] and others).

It should be noted that in zebrafish embryos, post-translational modifications of histones present just prior to zygotic genome activation are implicated in regulating the transcriptional profile at the onset of embryonic gene expression [59]. Thus, histone modifications may contribute to these parent-of-origin differences and the impact of paternally-derived chromatin on gene expression during genome activation may be evolutionarily conserved. Last, our study is the first to show that pharmacological manipulation of a normal metabolic pathway in a male leads to differential gene expression in his offspring by altering his sperm chromatin composition. Our experimental approach should provide a useful strategy for assessing the contribution of dietary and environmental factors, as well as therapeutic drugs, to inheritable changes of the sperm epigenome and consequently for offspring gene expression.

## Materials and Methods

### Mice and embryo collection

All procedures involving animals have been conducted as approved by the University of Pennsylvania Institutional Animal Care and Use Committee. Male mice from two different mouse models with compromised sperm chromatin (i.e., *Parg* gene disrupted mice (*Parg*(110)^−/−^ [52]) or PARP inhibitor-treated mice [29]) or the control males (wild-type/saline treated) were naturally mated to wild-type 129SVE (129S6/SvEvTac, Taconic) female mice and 2-cell embryos were collected. RNA from individual embryos was isolated and amplified using the WT-Ovation One-Direct RNA Amplification kit (NuGen Technologies). In the pharmacological mouse model excessive histone retention was induced by injecting wild-type males with PJ34 for six weeks prior to mating these males to wild-type females of the same inbred strain (129SVE).

### Gene expression microarrays

Gene expression profiles of individual embryos were generated by hybridization of the amplified cDNA to GeneChip Mouse Gene 1.0 ST arrays (Affymetrix). Gene expression data, as well as tiling microarray data (see below), were analyzed using Partek Genomics Suite software (Partek Inc., St. Louis, MO). Raw intensities were subjected to background correction, quantile normalization, log_2_ transformation and probe set summarization with the RMA (Robust Multichip Average) method. One-way ANOVA analyses of genotype and within genotypes was performed for pair-wise comparisons of expression data and the p-values adjusted for multiple testing using Benjamini-Hochberg correction for adjusted p-value calculation. Genes were identified as differentially expressed by ANOVA analyses if they had an adjusted p-value of P_adj_<0.1, which corresponds to a false discovery rate (FDR) of <10%.

To verify the *Parg*(110)^−/−^ and control microarray data, high throughput sequencing (HTS) on an Illumina HiSeq 2000 platform (Illumina) was performed. The overall similarity between the microarray and RNA sequencing data sets was calculated as the median correlation between each pair of matched microarray- and sequencing-based gene expression measurements (Fig. S4). The variance-stabilized expression values calculated by the DESeq package were used for HTS measurements, and the RMA-normalized values were used for the microarray measurements. Verification of PJ34/control microarray data was done using quantitative PCR employing custom qPCR arrays (Applied Biosystems, Taqman Array 96, Custom Format 16). The coefficient of variation across all samples in each group was calculated for every gene by calculating the ratio of the standard deviation (σ) to the mean (μ) (Cv = σ/μ) to identify highly variable genes.

### Two-cell embryo RNA sequencing and analysis

RNA-seq libraries were constructed from individual 2-cell embryos using amplified double stranded cDNA as the substrate in the Illumina bar-coded DNA-sequencing library preparation protocol (Illumina, San Diego, CA). The resulting sequencing reads were mapped to the mouse genome (mm9) using the RUM software package [60] (allowing up to 1 mismatch and excluding any reads that did not map uniquely) and differentially expressed genes were identified using the DESeq analysis package [61].

### Analyses of sperm histone DNA association using tiling arrays

After collecting the required number of embryos, male mice were sacrificed and cauda epididymal sperm collected. The degree of sperm chromatin condensation was analyzed by chromomycin A3 (CMA3) staining as described previously [29]. Mononuclease-sensitive fractions from sperm of individual mice were isolated using a swim-up technique with purification steps to eliminate all potentially contaminating somatic cells according to a well-established published method [4], [8], [33], [62], amplified using the Genomeplex Complete Whole Genome amplification (WGA2) kit (Sigma), and analyzed using GeneChip Mouse Promoter 1.0R tiling arrays (Affymetrix). To identify genes that abnormally remained bound by nucleosomes in the experimental group, mononucleosomal DNA fractions of sperm samples from individual sires (4 males in the PJ34 group, 10 males in *Parg*(110)^−/−^ and 9 control wild-type) were generated without pooling and gel-purified (Fig. S8A, B). Enrichment of histones in the mononucleosomal, soluble DNA fraction was confirmed by western blotting (Fig. S8C). All sperm samples of individual males were analyzed separately, using fragmented genomic sperm DNA for input subtraction. Tiling array data from all males in a given group (wild-type control, *Parg*(110)^−/−^, PJ34-treated) were used to identify sensitive gene regulatory regions associated with the soluble, i.e. nucleosomal fraction, using T-statistics (P<0.01) and a sliding window algorithm spanning 600 nucleotides. Nucleosomal enrichment of genomic regions in these single sample arrays was determined by pair-wise comparison with wild-type control data sets from individual control mice. A second data set was generated by combining array data from all *Parg*(110)^−/−^ samples compared to the pooled wild-type data set. Genomic regions affected by differential histone association gene promoter regions were identified by their differential association with the soluble mononucleosomal fraction in *Parg*(110)^−/−^ compared to wild-type control mice. Tiling microarray data were analyzed using Partek Genomics Suite software (Partek Inc., St. Louis, MO). Raw intensities were subjected to RMA normalization as described above for gene expression microarrays. Differential analysis between MNase-treated *Parg*(110)^−/−^, PJ34 treated and control samples were again carried out by computation of the T-statistic for each probe, followed by MAT (Model-based analysis of tiling arrays, [32]) to detect significant regions of enrichment in samples under consideration (p<0.01, and a sliding window of 600 nucleotides). Significant regions were then annotated with genes. To ascertain whether the genes affected by differential sperm histone association coded for specific cellular functions, gene ontology (GO) analyses were performed using DAVID [34], [35] (Fig. 3A). For these analyses, the gene lists were filtered for overlap of the detected significant regions (overlap>0%) to reduce the numbers of genes without introducing a bias to accommodate the 3,000 element input limit of the software. Genes that were both, differentially MNase sensitive in sperm and differentially expressed in 2-cell embryos (“2CEDE/MND” genes) were identified by pair-wise comparisons between the tiling array data from a single male with the 2CEDE gene lists from one of his offspring (level 1 list) or all of his offspring (level 2 list) or between whole groups (level 3 lists). All of these lists were compared with the tiling array data representing the differentially histone-associated genes in sperm of the respective father/wild-type control. Redundant hits of genes that were differentially expressed (DE) in two or more embryos were reduced to single hits for these analyses. Overlaps of lists containing DE genes with lists of genes that were differentially nucleosome associated genes in sperm were performed using a background of 19,472 genes that were interrogated by all array platforms (for the *Parg*(110)^−/−^ and PJ34-treated groups—for HTS data of *Parg*(110)^−/+^ embryo gene expression, the background was 20,018 genes due to the slightly larger number of genes commonly detected by HTS and tiling arrays). Overlaps of lists were identified using Partek software or the program VENNY [63]. Statistical significance of overlaps between tiling array and expression data was tested using Chi-squared tests with one degree of freedom and Yates's correction and by Pearson's Chi-squared test. These tests compare the predicted number of genes in the overlap (null hypothesis, i.e. coincidental overlap) with the observed number and determine confidence with which the observed number of genes in the overlap are higher (phi<0) or lower (phi>0) than the predicted number.

### Quantitative PCR

To verify microarray data of differential expression in 2-cell embryos from PJ34-treated fathers, quantitative PCR analyses were done using custom qPCR arrays (Applied Biosystems, Taqman Array 96, Custom Format 16) in a 96-well format according to the protocol provided by the manufacturer. For each of the embryos (9 from the PJ34-treated sire group, 7 from the control group) representing a sample, 40 ng of cDNA from the whole genome amplification reactions (see above) was used per experiment per sample. The analyses of nine differentially-expressed genes previously identified by microarrays, including *Hells/Lsh*, *Tet1*, *Suv39h*, and *Kdm4c*, and four control genes was performed. These genes were selected because they encode proteins involved in chromatin remodeling and modifying DNA and histones, and their differential expression at this early stage could have long-term effects on gene expression and further embryonic development. Four internal control genes were used: *Hprt*, *Eif1a*, *Gpd1l* and *Rn18s*, where the *Rn18s* was used for normalization. Targets and control genes were measured, each sample in triplicate, on the same plates. Analyses of cycle threshold (Ct) values were done using the Delta Delta Ct (ΔΔCt) method for each experiment. Assuming that PCR efficiency for the target was ∼100%, an approximation of fold-change expression was calculated as: Fold expression = 2^(ΔCt1-ΔCt2)^. The mean expression ratios of the genes in each experiment were calculated and the average value calculated for the six T-test analyses using Microsoft Excel was used to calculate p-values. As expected, transcript frequencies of control genes were not different between groups in microarray or qPCR analyses, confirming correct normalization of data and normal development of the embryos (Fig. S5).

All genomic and transcriptional data have been deposited in the NCBI GEO data base, accession numbers: GSE56254 (GSE56182, GSE56184, GSE56281, GSE5282), GSE55009.

## Supporting Information

Dataset S1 *Parg*(110)^−/−^ mouse model microarray data. Differentially nucleosome associated sperm genes in PargA, PargB, PargC (sires) sperm samples compared with the wildtype are listed according to relative histone enrichment (MAT(+)) or depletion (MAT(−)). Differentially expressed genes in 2-cell embryos obtained from these sires are listed by individual embryos (PargA1–3, PargB1–3, PargC1–3) and overlaps with differentially nucleosomal bound genes are listed. Data sets used to generate Figs. 2–6 are included in this MS Excel file, together with tables of complete GO-terms and Pearson correlation tables.
(XLSX)

Dataset S2 PJ34 mouse model microarray data. Similar to Dataset S1, differentially nucleosome associated sperm genes in PJ34A, PJ34B, PJ34C (sires) sperm samples compared with the wild-type are listed according to relative histone enrichment (MAT+) or depletion (MAT−). Differentially expressed genes in 2-cell embryos obtained from these sires are listed by individual embryos (PJ34A1–4, PJ34B1–4, PJ34C1–2) and overlaps with differentially nucleosomal bound genes are shown. Data sets used to generate Figs. 2–6 are included in Dataset S2 together with tables of complete GO-terms and Pearson correlation tables.
(XLSX)

Dataset S3 *Parg*(110)^−/−^ mouse model high throughput sequencing data. Similar to Dataset S1, differentially nucleosome associated sperm genes in *Parg*(110)^−/−^ sire sperm samples are compared with differentially expressed genes in 2-cell embryos, as determined by high throughput sequencing of single 2-cell embryo complete cDNA profiles obtained from these sires and overlaps are shown. This MS Excel file contains also the complete genome-wide sequencing data.
(XLSX)

Dataset S4 Data analyses and comparisons of the *Parg*(110)^−/−^ and PJ34 mouse models as well as data overlaps are in this file.
(XLSX)

Figure S1 Sperm samples are different between treatment groups and wild-type controls. To visualize differences between wild-type, *Parg*(110)^−/−^ and PJ34-treated males regarding their sperm MNase tiling array data sets, principal component analysis (PCA, PARTEK software package) was used as a simple eigenvector-based multivariate analyses routinely used to reveal the internal structure of the data that best explains the observed variance. PCA of the promoter tiling arrays hybridized with the MND fractions enriched in nucleosomal sperm DNA reveals segregation of fathers according to genotype. (A) PCA of MND fractions isolated from *Parg*(110)^−/−^ (n = 10) and wild-type control males (n = 9) indicates segregation between data sets.(B) Similar analysis of MND fractions isolated from PJ34 injected (n = 4, 10 mg/kg daily, over 10 weeks) and control males (n = 9), showing segregation between sperm tiling array data according to treatment group.
(PDF)

Figure S2 Promoter tiling arrays detect preferential sperm histone enrichment in defined genomic domains. (A) Normal enrichment of nucleosomes in promoter regions compared to intron or coding sequences (CDS) was detectable with high confidence (P-values essentially approaching “0”, i.e. P≪0.0001) in all sperm sample MNase fractionated DNA preparations corrected by input genomic DNA. The wild-type was comprised of 9 individual sperm samples (Wildtype), the individual males used for father-offspring analyses are all shown (PargA, PargB, PargC, as well as PJ34A, PJ34B and PJ34C). (B) Positive association of nucleosome enrichment with high, intermediate and low density CpG [1] content of the DNA was detected in all data sets (P≪0.0001, see above). (C) DNA methylation was inversely correlated with nucleosome association in all sperm samples (P≪0.0001).
(PDF)

Figure S3 Variance analyses of gene expression did not reveal major differences in gene expression between 2-cell embryos from males with altered PAR metabolism and corresponding embryos from wild-type untreated control males. The coefficient of variation (Cv, i.e., the ratio of the standard deviation (σ) to the mean (μ) (Cv = σ/μ) to identify highly variable genes) of all genes interrogated by the microarray analyses was calculated for each gene, followed by sorting of genes according to Cv value. The resulting graph from *Parg*(110)^+/−^, PJ34 and control embryos are nearly overlapping with similar mean Cv values but due to the large number of data the small difference is significant as determined by ANOVA analyses (p<0.05). Mean values are indicated.
(PDF)

Figure S4 Correlation between microarray and next-generation sequencing analyses results of Parg(110)^+/−^ expression for confirmation of the *Parg*(110)^+/−^ 2-cell embryo expression microarray data. The overall similarity between the microarray and RNA sequencing data sets was calculated as the median correlation between each pair of matched microarray- and sequencing-based gene expression measurements. The variance-stabilized expression values calculated by the DESeq package were used for HTS measurements, and the RMA-normalized values were used for the microarray measurements. The mean Pearson correlation is ∼72%. The whole-dataset comparison was plotted with 18 independent samples (9 wild-type control+9 *Parg*(110)^−/+^ 2CE).
(PDF)

Figure S5 Custom PCR arrays confirming differential expression of select genes identified as differentially-expressed in microarrays of the PJ34/control 2-cell embryo group. (A) Nine 2-cell embryos per treatment group were subjected to qPCR analysis of genes previously identified as differentially expressed in microarrays. The dotted line indicates 18S RNA normalization and asterisks indicate statistically significant (*, p<0.05, Student's t-test) or highly significant (**, p<0.001) differences from 2-cell embryos of saline treated controls. (B) Scatter blots of microarray data show variance of expression in 5 differentially-expressed genes according to the father's treatment group (red dots: PJ34, blue dots: saline), consistent with individual sperm variation (see also Fig. 2C). Besides 18S RNA (*Rn18s*), three control genes that were unaltered in the microarrays were included (*Eif1a*, *Gpd1l* and *Hprt1*), which were previously identified as unaltered in the microarrays, depending on the PJ34 (red) or saline (blue) treatment of their fathers.
(PDF)

Figure S6 Representative examples of genes that demonstrate correlations of aberrant sperm histone association with differential embryonic gene expression. **PJ34 model:** (A) *Rpl15* (0.4-fold expression in PJ34A progeny, false discovery rate (FDR) = 0.03), (B) *Pou5f1* (also known as *Oct4*, abnormally elevated sperm histone retention in PJ34A and PJ34C but 0.3-fold expression in PJ34C progeny only, FDR = 0.05), (C) *Ctla4* (3.6-fold increased expression in PJ34A progeny only, FDR = 0.02); ***Parg*** **(110)^−/−^ model** (D) *Vmn2r112* (reduced histone retention in PargB versus the wildtype, note the overall already low/absent histone retention in the wildtype except for a marked area, where nucleosomes are normally retained in low concentrations in the wildtype but depleted in sample PargB, 1.7-fold expression in PargB progeny, FDR = 0.08), (E) *Sco1* (reduced histone retention in samples PargA–C, with 11.3-fold expression, FDR = 0.04 in PargA progeny only), (F) *EnoxI* (2.34-fold expression in PargB, FDR = 0.06).
(PDF)

Figure S7 Sperm histone association in wild-type (WT con), *Parg*(110)^−/−^ (PargA–C) and PARP inhibitor treated (PJ34A–C) males across a section of chromosome 9 shows that results of the tiling arrays were consistent between samples and experiments. Positive bars in the four top tracks from individual MND analyses of *Parg* KO males show histone enrichment in sperm relative to the genomic input control. A predominant absence of histones in distinct areas is indicated by negative bars, i.e., higher values in the genomic input fraction. Red boxes indicate the locations of two olfactory receptor (*Olfr*) gene clusters with their relatively low histone content (predominance of negative bars); the blue box indicates a cluster of mostly housekeeping genes, including for example the *Dnmt1* gene, and that has a comparatively higher normal abundance of histones in sperm (predominantly positive bars). Note that GC content is positively correlated with gene density and nucleosome enrichment in and that *Olfr* gene clusters have overall relatively low GC content and low nucleosome association.
(PDF)

Figure S8 Sperm MNDS isolation and analyses. (A) Flowchart of sperm MNDS isolation procedure. (B) After limited MNase digestion of 1 million sperm from an individual mouse the supernatant contains low molecular weight histone-associated DNA of ∼150 base pairs, i.e., the equivalent of DNA bound by a single nucleosome (lane 1, red arrow), whereas the pellet retains mostly MNase-resistant DNA (lane 2, blue arrow). (C) Histone H3 immunoblot analysis of MNase-soluble and -insoluble sperm fractions demonstrates histone enrichment in the soluble fraction. After MNase digestion the supernatant (lane 2, supernatant equivalent to 3×10^6^ sperm was loaded) contains more histone H3 protein than the pellet of the same reaction (lane 3, equivalent to 3×10^6^ sperm was loaded). Lane 1 contains lysate of 5×10^5^ undigested sperm from the same animal.
(PDF)

Figure S9 (Relevant to Fig. 4): P-values of Pearson (uncorrected) and Yates (corrected) Chi-squared tests to determine the significance of overlaps of the lists of genes that were differentially histone associated in sperm samples of the sires compared to controls (Parg A–C and PJ34A–C, panels a–f) with the lists of genes that were differentially expressed in at least one of the 3 or 4 offspring 2-cell embryos from these sires (*Parg*(110)^−/−^: A 1–3, B 1–3, C 1–3, panels a–d; and for PJ34: A1–4, B1–4, C1–2, panels e, f) A genetic background of 19,472 genes interrogated by the microarrays and 20,018 genes interrogated by the tiling arrays and sequencing platforms was used for the calculations. MAT−: genes with abnormally low sperm histone retention in *Parg*(110)^−/−^ or PJ34 sperm compared to controls. MAT+: genes with abnormally elevated sperm histone retention compared to controls genes. MAT−/+: combination of MAT− and MAT+ lists. P-values resulting from Yates or Pearson are highlighted in mauve if P≤0.05, i.e., the overlaps were significant. The P-value denotes the confidence with which the null-hypothesis can be dismissed that the overlap between the list of genes with abnormal histone association in the sire with the list of genes that are DE in the offspring could be predicted by statistical probability, i.e. coincidence. Because these Chi-squared tests are two-directional, an inverse correlation can be detected by calculation of the phi value where Phi>0 indicates a negative correlation. P-values indicating a negative correlation are in red font; Panels a, b: *Parg*(110) group of fathers and offspring embryos, microarray expression analyses with either Yates' chi-squared test (a) or Pearson's (b); Panels c, d: *Parg*(110) group, high throughput sequencing of 2CE gene expression, using Yates' (c) or Pearson's (d) chi-squared test; Panels e, f: PJ34 group of fathers and offspring embryos, Yates' (e) or Pearson's chi-squared test (f). Note that mainly overlaps between genes with lower histone retention in sperm and differentially expressed genes in offspring embryos are significant with P≤0.05.
(PDF)
