# Supplementary figures and images for: Paternal Poly (ADP-ribose) Metabolism Modulates Retention of Inheritable Sperm Histones and Early Embryonic Gene Expression
Source: PLoS Genet. 2014 May 8;10(5):e1004317. doi: 10.1371/journal.pgen.1004317 (PMC4014456; doi:10.1371/journal.pgen.1004317)

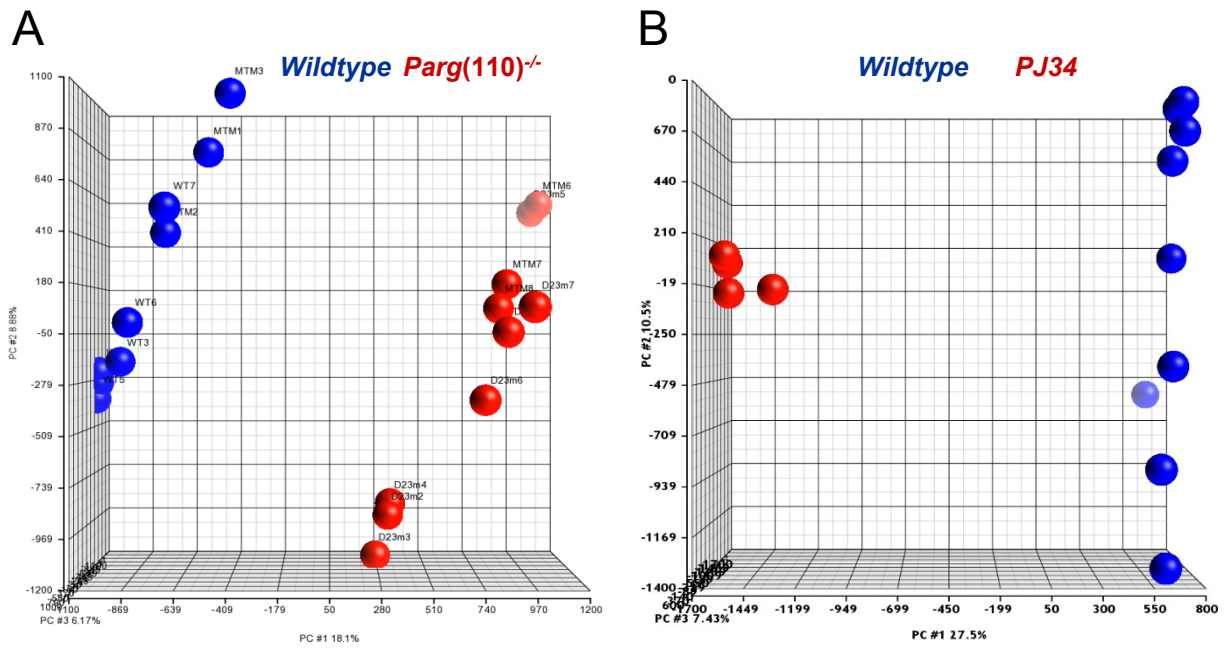

Figure S1

Supplement: Figure S1 — Sperm samples are different between treatment groups and wild-type controls. To visualize differences between wild-type, Parg(110)−/− and PJ34-treated males regarding their sperm MNase tiling array data sets, principal component analysis (PCA, PARTEK software package) was used as a simple eigenvector-based multivariate analyses routinely used to reveal the internal structure of the data that best explains the observed variance. PCA of the promoter tiling arrays hybridized with the MND fractions enriched in nucleosomal sperm DNA reveals segregation of fathers according to genotype. (A) PCA of MND fractions isolated from Parg(110)−/− (n = 10) and wild-type control males (n = 9) indicates segregation between data sets.(B) Similar analysis of MND fractions isolated from PJ34 injected (n = 4, 10 mg/kg daily, over 10 weeks) and control males (n = 9), showing segregation between sperm tiling array data according to treatment group. (PDF) [file pgen.1004317.s005.pdf]

A

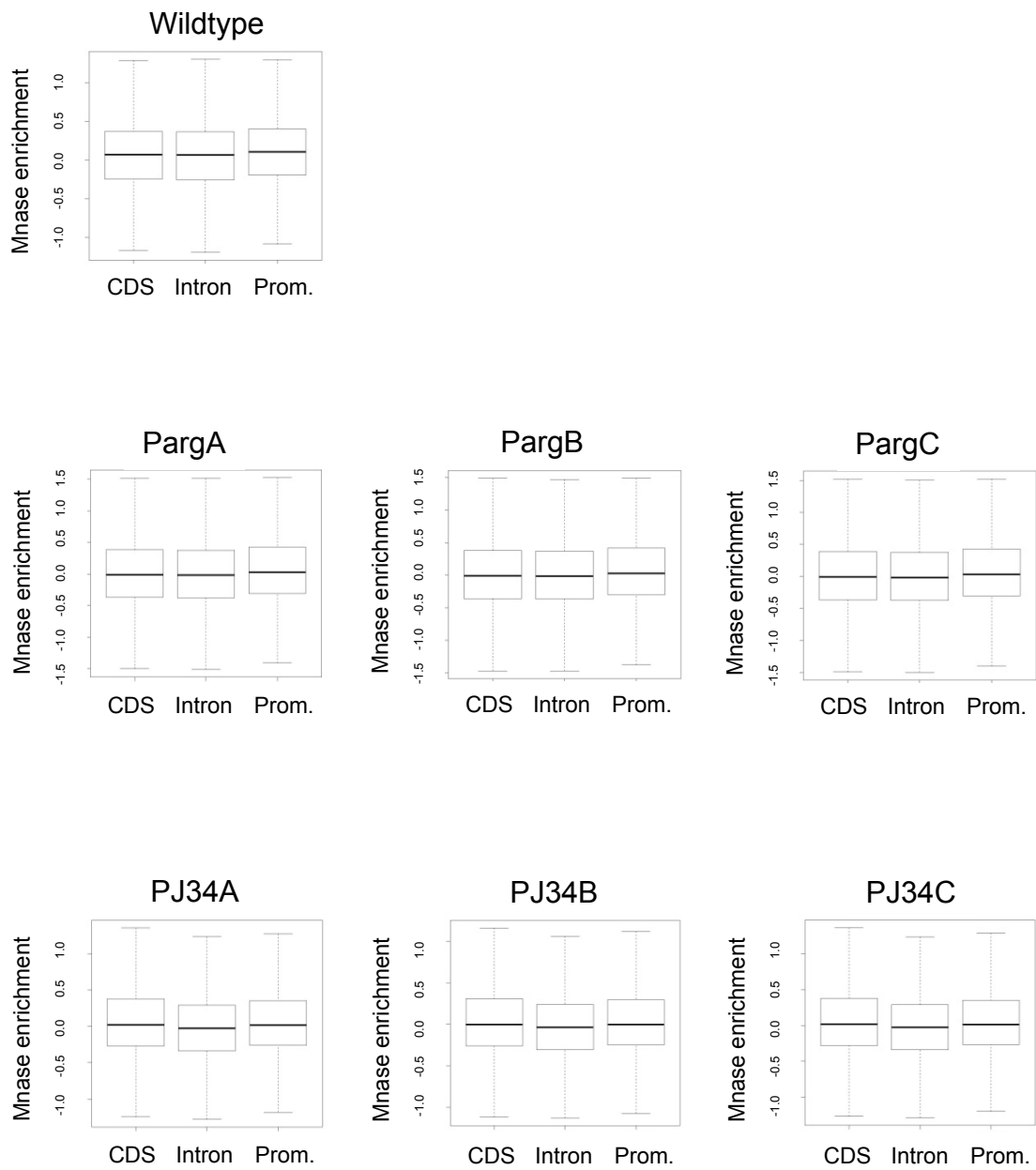

( $P < 0.0001$  in all samples)

Figure S2

**B**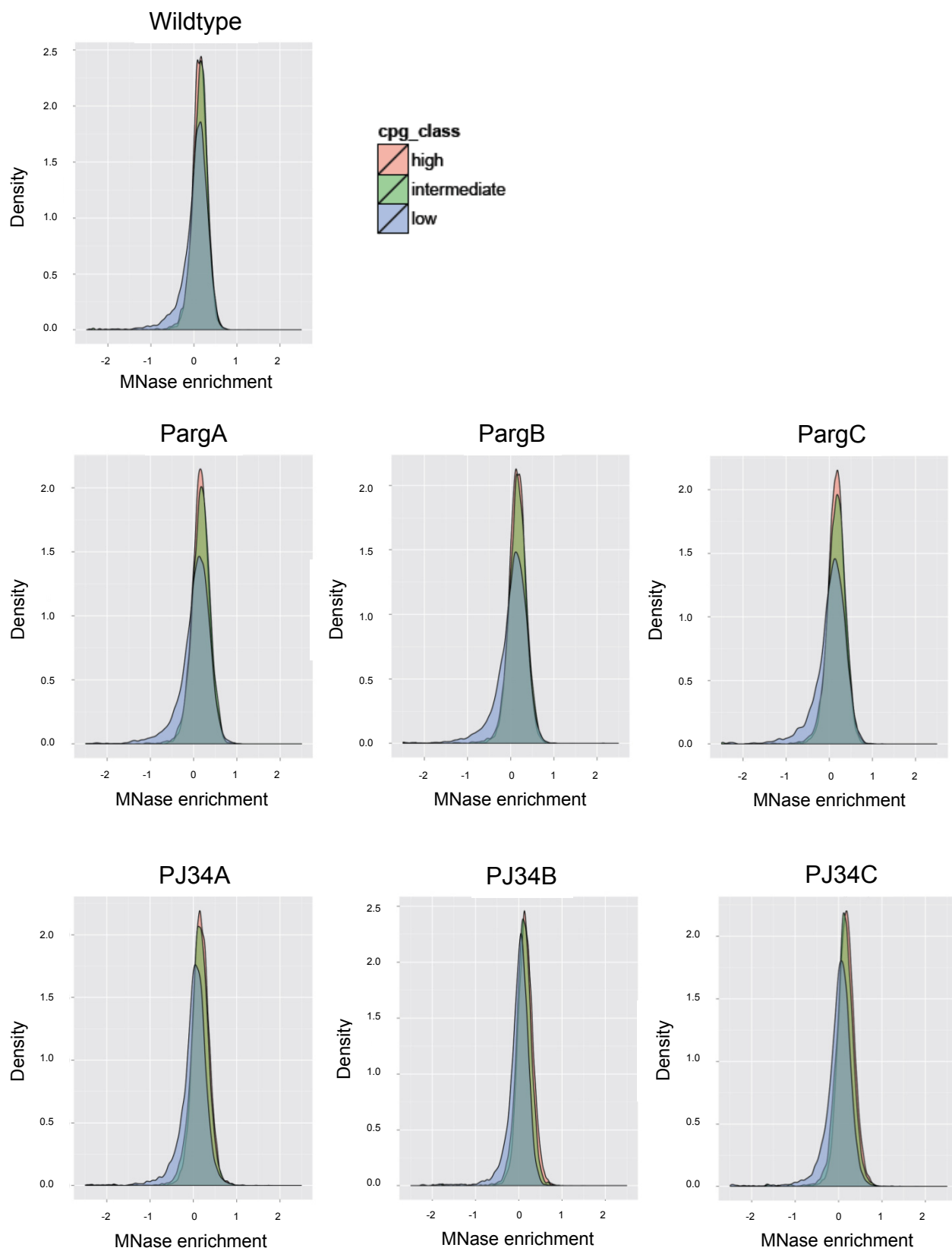**Figure S2**

( $P < 0.0001$  in all samples)

C

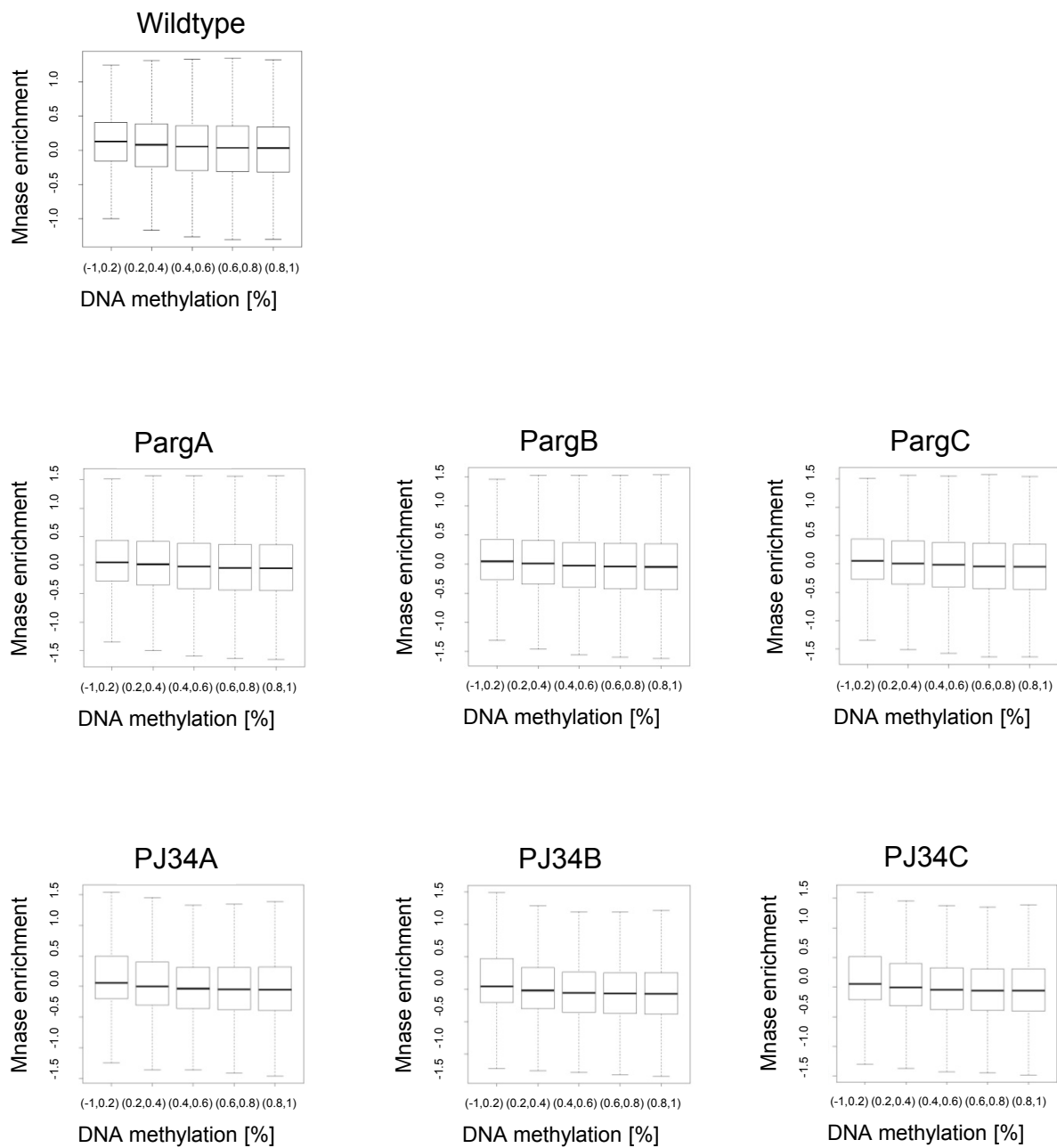

(P <<0.0001 in all samples)

Figure S2

Supplement: Figure S2 — Promoter tiling arrays detect preferential sperm histone enrichment in defined genomic domains. (A) Normal enrichment of nucleosomes in promoter regions compared to intron or coding sequences (CDS) was detectable with high confidence (P-values essentially approaching “0”, i.e. P≪0.0001) in all sperm sample MNase fractionated DNA preparations corrected by input genomic DNA. The wild-type was comprised of 9 individual sperm samples (Wildtype), the individual males used for father-offspring analyses are all shown (PargA, PargB, PargC, as well as PJ34A, PJ34B and PJ34C). (B) Positive association of nucleosome enrichment with high, intermediate and low density CpG [1] content of the DNA was detected in all data sets (P≪0.0001, see above). (C) DNA methylation was inversely correlated with nucleosome association in all sperm samples (P≪0.0001). (PDF) [file pgen.1004317.s006.pdf]

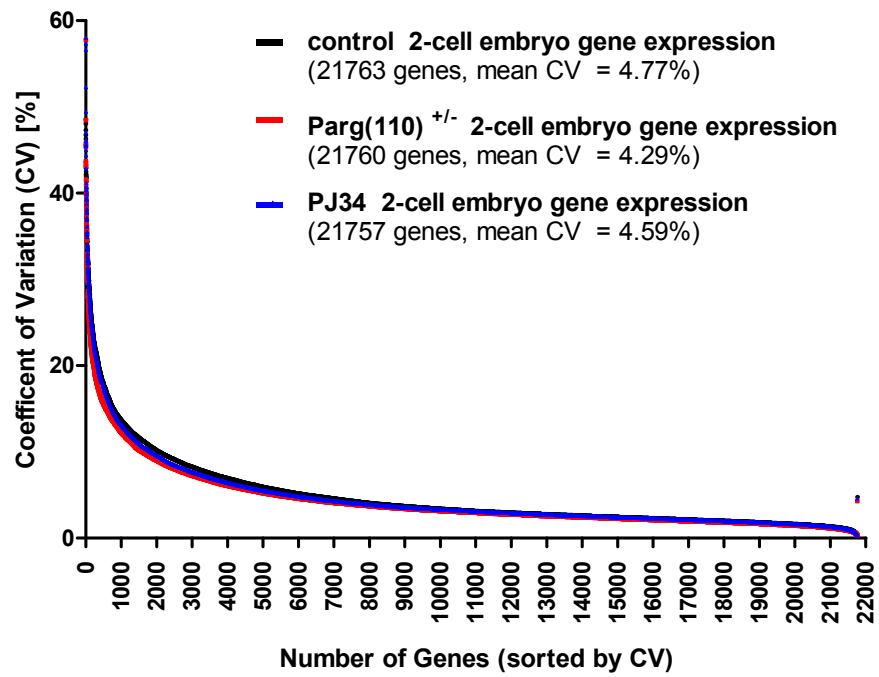

Figure S3

Supplement: Figure S3 — Variance analyses of gene expression did not reveal major differences in gene expression between 2-cell embryos from males with altered PAR metabolism and corresponding embryos from wild-type untreated control males. The coefficient of variation (Cv, i.e., the ratio of the standard deviation (σ) to the mean (μ) (Cv = σ/μ) to identify highly variable genes) of all genes interrogated by the microarray analyses was calculated for each gene, followed by sorting of genes according to Cv value. The resulting graph from Parg(110)+/−, PJ34 and control embryos are nearly overlapping with similar mean Cv values but due to the large number of data the small difference is significant as determined by ANOVA analyses (p<0.05). Mean values are indicated. (PDF) [file pgen.1004317.s007.pdf]

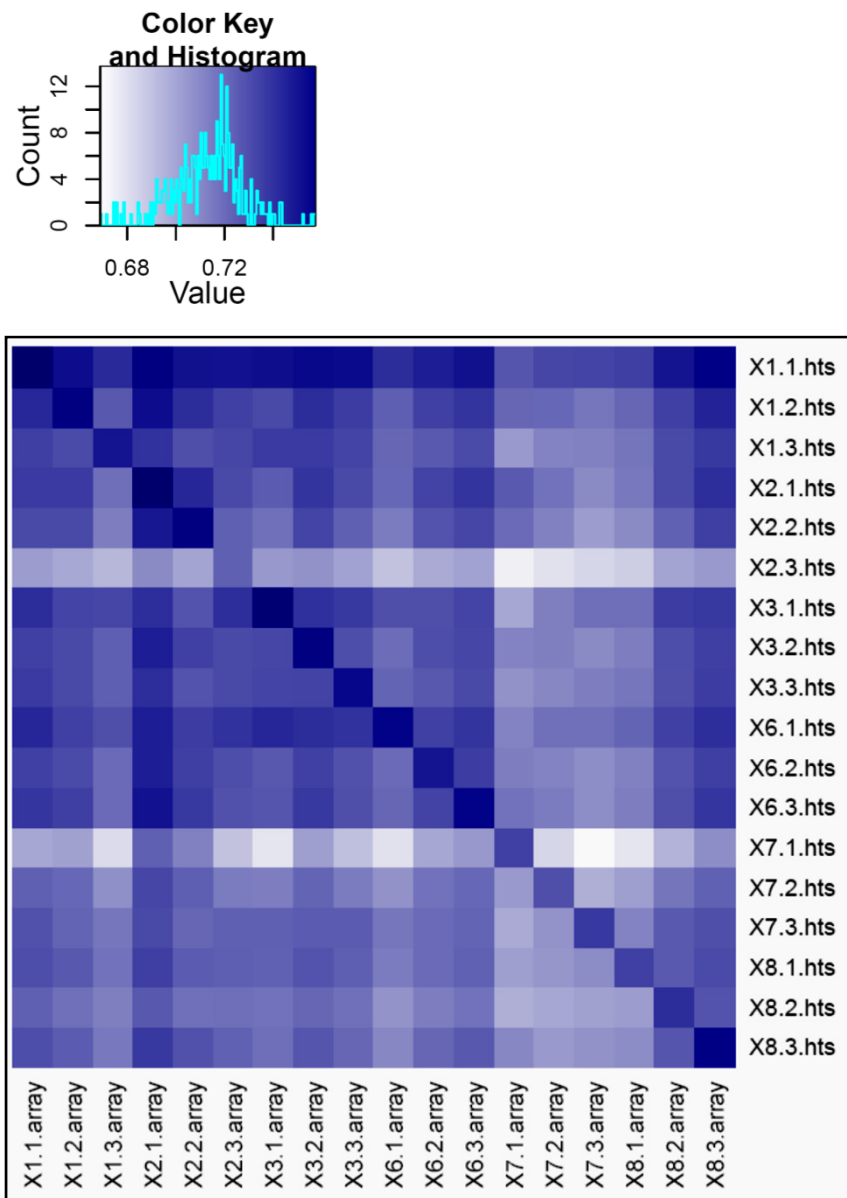

Figure S4

Supplement: Figure S4 — Correlation between microarray and next-generation sequencing analyses results of Parg(110)+/− expression for confirmation of the Parg(110)+/− 2-cell embryo expression microarray data. The overall similarity between the microarray and RNA sequencing data sets was calculated as the median correlation between each pair of matched microarray- and sequencing-based gene expression measurements. The variance-stabilized expression values calculated by the DESeq package were used for HTS measurements, and the RMA-normalized values were used for the microarray measurements. The mean Pearson correlation is ∼72%. The whole-dataset comparison was plotted with 18 independent samples (9 wild-type control+9 Parg(110)−/+ 2CE). (PDF) [file pgen.1004317.s008.pdf]

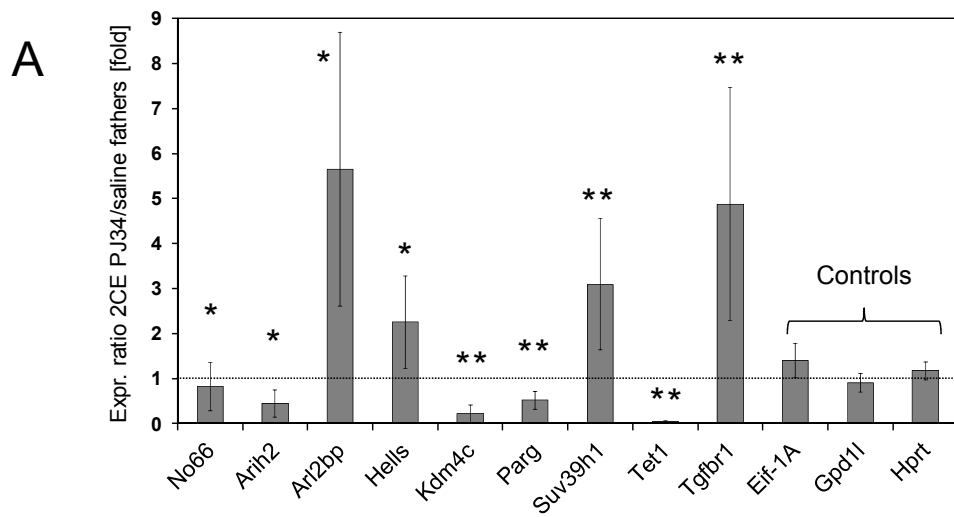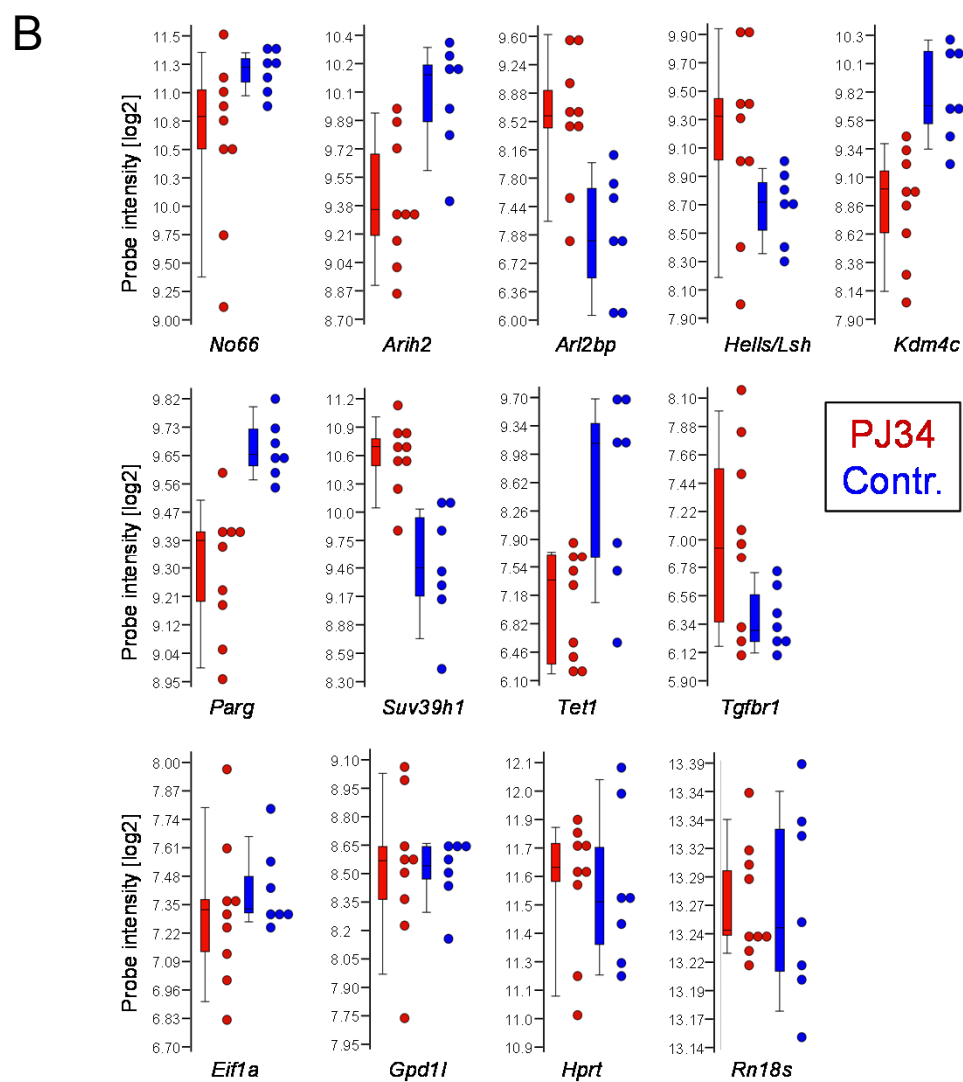

Figure S5

Supplement: Figure S5 — Custom PCR arrays confirming differential expression of select genes identified as differentially-expressed in microarrays of the PJ34/control 2-cell embryo group. (A) Nine 2-cell embryos per treatment group were subjected to qPCR analysis of genes previously identified as differentially expressed in microarrays. The dotted line indicates 18S RNA normalization and asterisks indicate statistically significant (*, p<0.05, Student's t-test) or highly significant (**, p<0.001) differences from 2-cell embryos of saline treated controls. (B) Scatter blots of microarray data show variance of expression in 5 differentially-expressed genes according to the father's treatment group (red dots: PJ34, blue dots: saline), consistent with individual sperm variation (see also Fig. 2C). Besides 18S RNA (Rn18s), three control genes that were unaltered in the microarrays were included (Eif1a, Gpd1l and Hprt1), which were previously identified as unaltered in the microarrays, depending on the PJ34 (red) or saline (blue) treatment of their fathers. (PDF) [file pgen.1004317.s009.pdf]

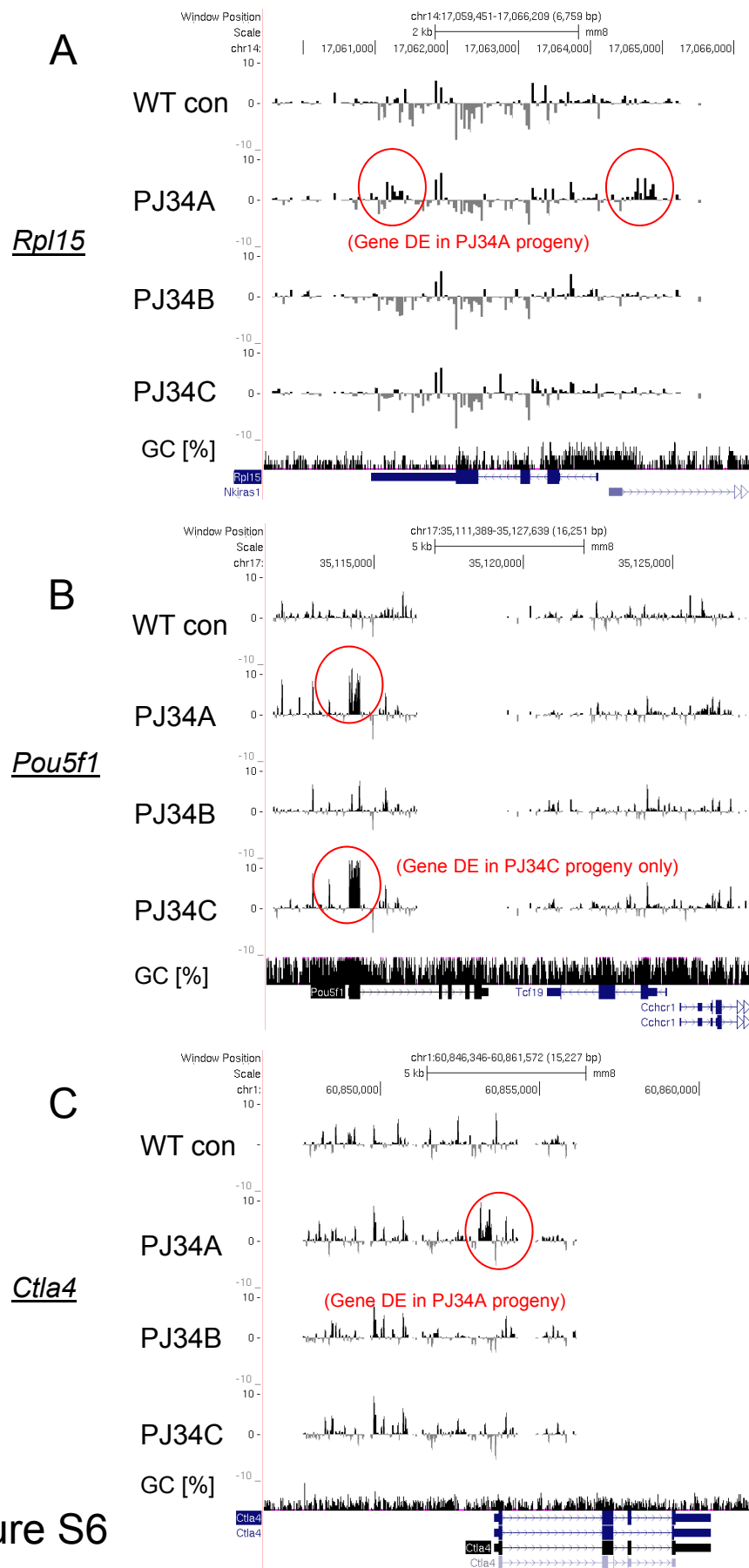

Figure S6

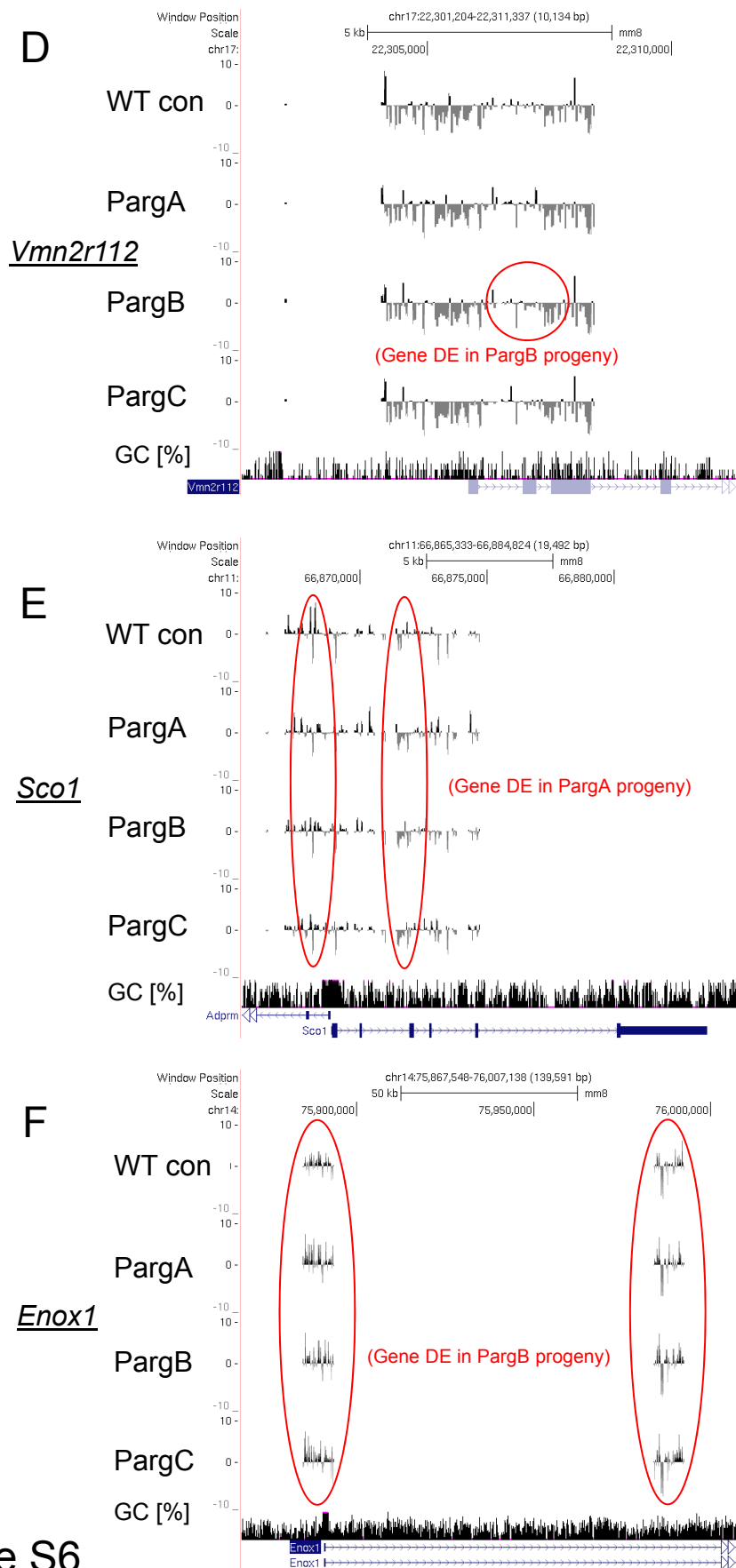

Figure S6

Supplement: Figure S6 — Representative examples of genes that demonstrate correlations of aberrant sperm histone association with differential embryonic gene expression. PJ34 model: (A) Rpl15 (0.4-fold expression in PJ34A progeny, false discovery rate (FDR) = 0.03), (B) Pou5f1 (also known as Oct4, abnormally elevated sperm histone retention in PJ34A and PJ34C but 0.3-fold expression in PJ34C progeny only, FDR = 0.05), (C) Ctla4 (3.6-fold increased expression in PJ34A progeny only, FDR = 0.02); Parg (110)−/− model (D) Vmn2r112 (reduced histone retention in PargB versus the wildtype, note the overall already low/absent histone retention in the wildtype except for a marked area, where nucleosomes are normally retained in low concentrations in the wildtype but depleted in sample PargB, 1.7-fold expression in PargB progeny, FDR = 0.08), (E) Sco1 (reduced histone retention in samples PargA–C, with 11.3-fold expression, FDR = 0.04 in PargA progeny only), (F) EnoxI (2.34-fold expression in PargB, FDR = 0.06). (PDF) [file pgen.1004317.s010.pdf]

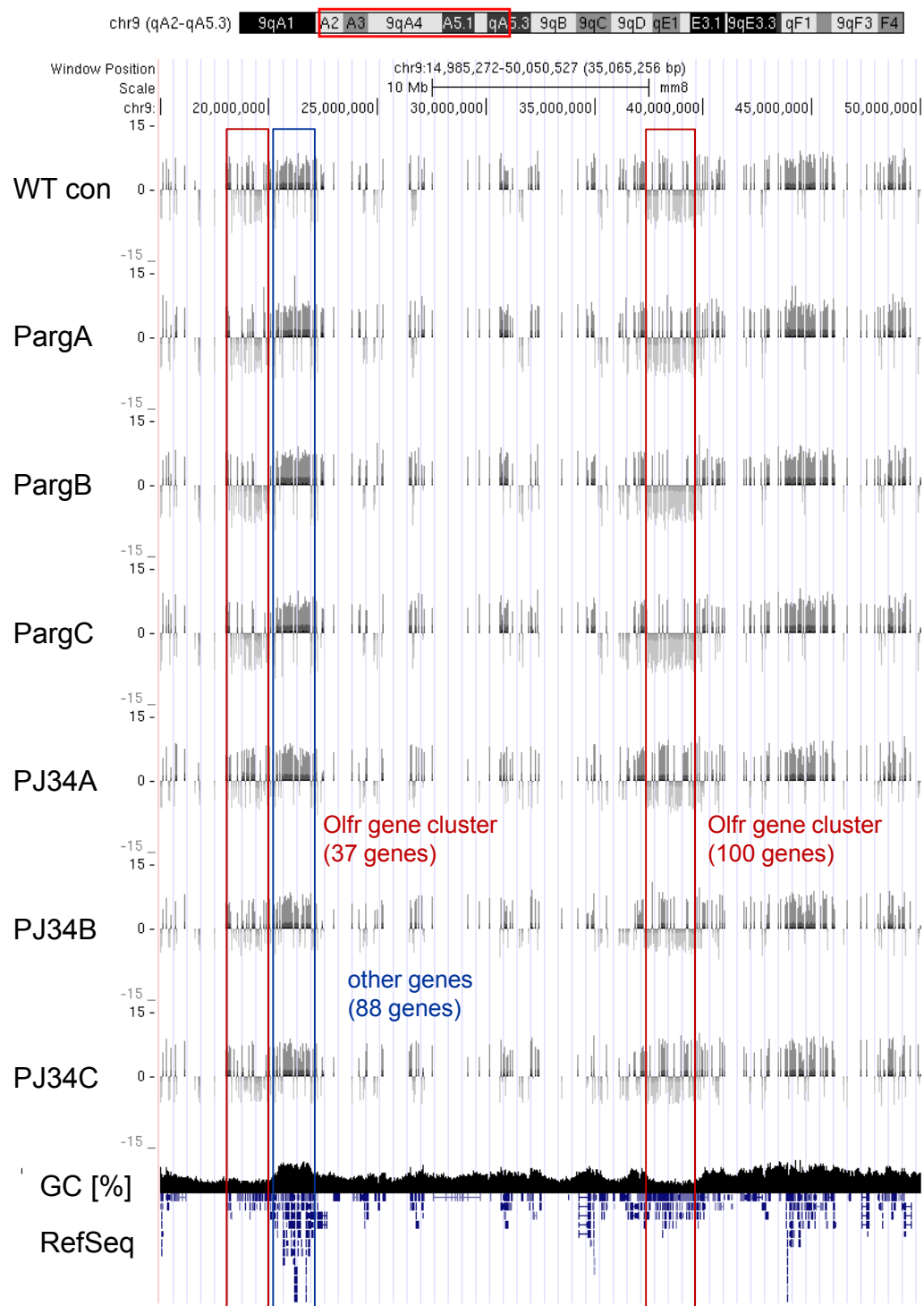

Figure S7

Supplement: Figure S7 — Sperm histone association in wild-type (WT con), Parg(110)−/− (PargA–C) and PARP inhibitor treated (PJ34A–C) males across a section of chromosome 9 shows that results of the tiling arrays were consistent between samples and experiments. Positive bars in the four top tracks from individual MND analyses of Parg KO males show histone enrichment in sperm relative to the genomic input control. A predominant absence of histones in distinct areas is indicated by negative bars, i.e., higher values in the genomic input fraction. Red boxes indicate the locations of two olfactory receptor (Olfr) gene clusters with their relatively low histone content (predominance of negative bars); the blue box indicates a cluster of mostly housekeeping genes, including for example the Dnmt1 gene, and that has a comparatively higher normal abundance of histones in sperm (predominantly positive bars). Note that GC content is positively correlated with gene density and nucleosome enrichment in and that Olfr gene clusters have overall relatively low GC content and low nucleosome association. (PDF) [file pgen.1004317.s011.pdf]

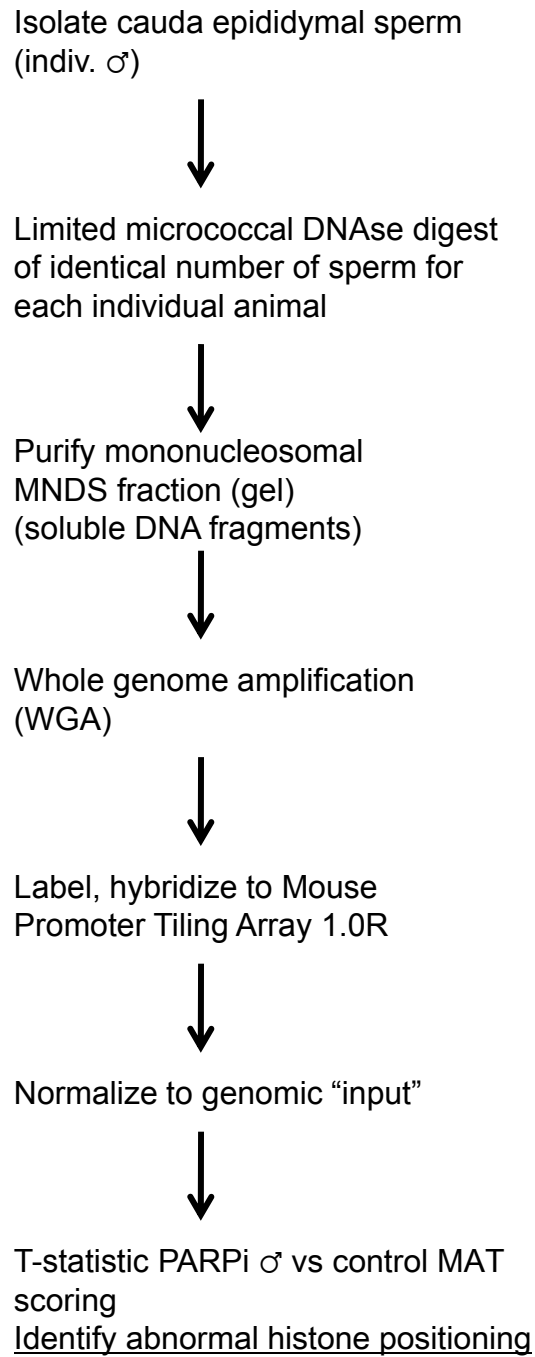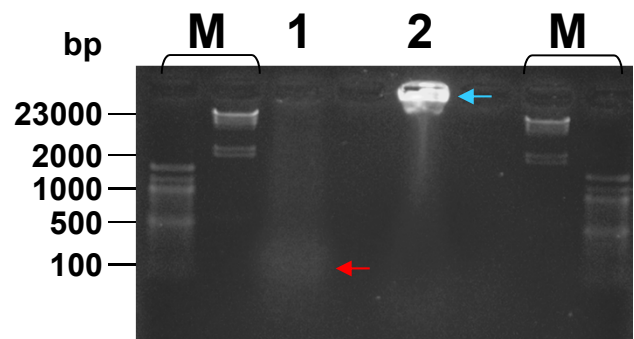

B

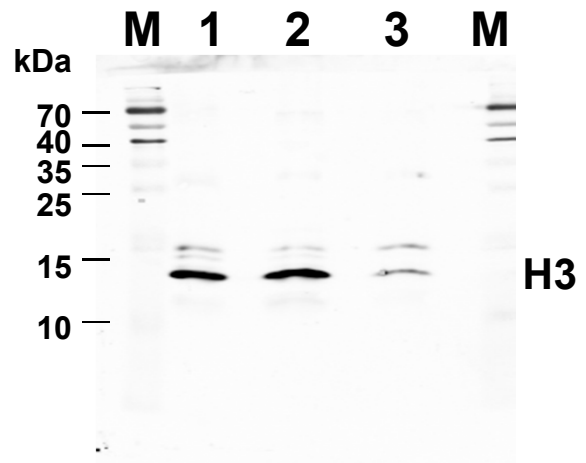

C

A

Figure S8

Supplement: Figure S8 — Sperm MNDS isolation and analyses. (A) Flowchart of sperm MNDS isolation procedure. (B) After limited MNase digestion of 1 million sperm from an individual mouse the supernatant contains low molecular weight histone-associated DNA of ∼150 base pairs, i.e., the equivalent of DNA bound by a single nucleosome (lane 1, red arrow), whereas the pellet retains mostly MNase-resistant DNA (lane 2, blue arrow). (C) Histone H3 immunoblot analysis of MNase-soluble and -insoluble sperm fractions demonstrates histone enrichment in the soluble fraction. After MNase digestion the supernatant (lane 2, supernatant equivalent to 3×10^6^ sperm was loaded) contains more histone H3 protein than the pellet of the same reaction (lane 3, equivalent to 3×10^6^ sperm was loaded). Lane 1 contains lysate of 5×10^5^ undigested sperm from the same animal. (PDF) [file pgen.1004317.s012.pdf]
