# Supplementary material for: Paternal Poly (ADP-ribose) Metabolism Modulates Retention of Inheritable Sperm Histones and Early Embryonic Gene Expression
Source: PLoS Genet. 2014 May 8;10(5):e1004317. doi: 10.1371/journal.pgen.1004317 (PMC4014456; doi:10.1371/journal.pgen.1004317)
Supplement: Figure S9 — (Relevant to Fig. 4): P-values of Pearson (uncorrected) and Yates (corrected) Chi-squared tests to determine the significance of overlaps of the lists of genes that were differentially histone associated in sperm samples of the sires compared to controls (Parg A–C and PJ34A–C, panels a–f) with the lists of genes that were differentially expressed in at least one of the 3 or 4 offspring 2-cell embryos from these sires (Parg(110)−/−: A 1–3, B 1–3, C 1–3, panels a–d; and for PJ34: A1–4, B1–4, C1–2, panels e, f) A genetic background of 19,472 genes interrogated by the microarrays and 20,018 genes interrogated by the tiling arrays and sequencing platforms was used for the calculations. MAT−: genes with abnormally low sperm histone retention in Parg(110)−/− or PJ34 sperm compared to controls. MAT+: genes with abnormally elevated sperm histone retention compared to controls genes. MAT−/+: combination of MAT− and MAT+ lists. P-values resulting from Yates or Pearson are highlighted in mauve if P≤0.05, i.e., the overlaps were significant. The P-value denotes the confidence with which the null-hypothesis can be dismissed that the overlap between the list of genes with abnormal histone association in the sire with the list of genes that are DE in the offspring could be predicted by statistical probability, i.e. coincidence. Because these Chi-squared tests are two-directional, an inverse correlation can be detected by calculation of the phi value where Phi>0 indicates a negative correlation. P-values indicating a negative correlation are in red font; Panels a, b: Parg(110) group of fathers and offspring embryos, microarray expression analyses with either Yates' chi-squared test (a) or Pearson's (b); Panels c, d: Parg(110) group, high throughput sequencing of 2CE gene expression, using Yates' (c) or Pearson's (d) chi-squared test; Panels e, f: PJ34 group of fathers and offspring embryos, Yates' (e) or Pearson's chi-squared test (f). Note that mainly overlaps between genes with lo [file pgen.1004317.s013.pdf]

a

2CEDE (Yates)	Lower histone retention (MAT-)			Elevated histone retention (MAT+)			Lower and elevated HR (MAT-/+)		
	PARG A	PARG B	PARG C	PARG A	PARG B	PARG C	PARG A	PARG B	PARG C
A 1-3	0.051	0.275	0.105	0.420	0.920	0.663	0.639	0.777	0.146
B 1-3	0.063	0.047	0.088	0.603	0.024	0.888	0.229	0.920	0.159
C 1-3	0.169	0.131	0.060	0.399	1.000	0.718	0.251	0.327	0.083
All 2CE	0.026	0.005	0.009	0.212	0.431	0.823	0.663	0.128	0.016

b

2CEDE (Pearson)	Lower histone retention (MAT-)			Elevated histone retention (MAT+)			Lower and elevated HR (MAT-/+)		
	PARG A	PARG B	PARG C	PARG A	PARG B	PARG C	PARG A	PARG B	PARG C
A 1-3	0.041	0.238	0.088	0.368	0.823	0.597	0.584	0.729	0.128
B 1-3	0.055	0.041	0.078	0.560	0.021	0.823	0.888	0.210	0.146
C 1-3	0.147	0.113	0.050	0.357	1.000	0.663	0.227	0.299	0.073
All 2CE	0.023	0.004	0.008	0.195	0.406	0.791	0.639	0.119	0.014

c

HTS 2CE (Yates)	Lower histone retention (MAT-)			Elevated histone retention (MAT+)			Lower and elevated HR (MAT-/+)		
	PARG A	PARG B	PARG C	PARG A	PARG B	PARG C	PARG A	PARG B	PARG C
A 1-3	0.017	0.584	0.740	0.823	0.603	0.517	0.046	0.219	0.357
B 1-3	0.024	0.026	0.222	0.012	0.348	0.863	0.005	0.003	0.306
C 1-3	0.752	0.560	0.708	0.752	0.185	0.590	1.000	0.920	0.920
All 2CE	0.017	0.029	0.219	0.166	0.560	0.823	0.011	0.004	0.190

d

HTS 2CE (Pearson)	Lower histone retention (MAT-)			Elevated histone retention (MAT+)			Lower and elevated HR (MAT-/+)		
	PARG A	PARG B	PARG C	PARG A	PARG B	PARG C	PARG A	PARG B	PARG C
A 1-3	0.012	0.484	0.624	0.689	0.493	0.417	0.034	0.176	0.297
B 1-3	0.018	0.020	0.182	0.008	0.288	0.764	0.004	0.003	0.264
C 1-3	0.647	0.467	0.597	0.632	0.137	0.488	1.000	1.000	1.000
All 2CE	0.013	0.023	0.190	0.140	0.502	0.764	0.009	0.003	0.168

e

2CEDE (Yates)	Lower histone retention (MAT-)			Elevated histone retention (MAT+)			Lower and elevated HR (MAT-/+)		
	PJ34A	PJ34B	PJ34C	PJ34A	PJ34B	PJ34C	PJ34A	PJ34B	PJ34C
A 1-4	0.015	0.1	0.025	0.133	0.729	1	0.009	0.242	0.04
B 1-4	0.085	0.446	0.057	0.174	0.036	1	0.188	0.35	0.462
C 1-2	1	0.286	0.617	0.764	1	0.718	0.92	0.454	0.92
All 2CE	0.027	0.092	0.007	0.086	0.265	0.862	0.023	0.159	0.054

f

2CEDE (Pearson)	Lower histone retention (MAT-)			Elevated histone retention (MAT+)			Lower and elevated HR (MAT-/+)		
	PJ34A	PJ34B	PJ34C	PJ34A	PJ34B	PJ34C	PJ34A	PJ34B	PJ34C
A 1-4	0.013	0.089	0.022	0.119	0.689	0.92	0.008	0.224	0.036
B 1-4	0.074	0.41	0.049	0.154	0.029	1	0.17	0.322	0.431
C 1-2	0.92	0.247	0.56	0.689	0.92	0.639	0.862	0.406	0.841
All 2CE	0.024	0.083	0.009	0.079	0.244	0.841	0.021	0.149	0.05

Figure S9
